# Effect of the COVID-19 pandemic and international travel ban on elephant tourist camp management in northern Thailand

**DOI:** 10.3389/fvets.2022.1038855

**Published:** 2022-12-02

**Authors:** Jarawee Supanta, Janine L. Brown, Pakkanut Bansiddhi, Chatchote Thitaram, Veerasak Punyapornwithaya, Jaruwan Khonmee

**Affiliations:** ^1^Department of Veterinary Bioscience and Veterinary Public Health, Faculty of Veterinary Medicine, Chiang Mai University, Chiang Mai, Thailand; ^2^Center of Elephant and Wildlife Health, Chiang Mai University Animal Hospital, Chiang Mai, Thailand; ^3^Center for Species Survival, Smithsonian Conservation Biology Institute, Front Royal, VA, United States; ^4^Department of Companion Animal and Wildlife Clinic, Faculty of Veterinary Medicine, Chiang Mai University, Chiang Mai, Thailand; ^5^Department of Food Animal Clinic, Faculty of Veterinary Medicine, Chiang Mai University, Chiang Mai, Thailand

**Keywords:** Asian elephant, tourist camp, management, welfare, COVID-19, Thailand

## Abstract

The COVID-19 pandemic has had a significant impact on the tourism industry, especially in Thailand. Starting in April 2020, the Thai government banned international travel and all elephant tourist camps closed. A wide variety of management changes were implemented because of the lack of income from tourists. This study surveyed 30 camps that cared for >400 elephants in northern Thailand to obtain information on camp, elephant, and mahout management during the COVID-19 pandemic from April 2020 to 2022 compared to the year before. The survey consisted of questionnaires that interviewed elephant camp owners, managers, veterinarians, and mahouts, and captured information on changes in camp operations, including numbers of tourists, elephants and mahouts, elephant and mahout activities, and veterinary care. Results revealed significant changes in camp structure, elephant work activities and general care. Staff layoffs led to a decrease in the ratio of mahouts to elephants from 1:1 to 1:2. Elephant activities, distance walked, and amounts of food were reduced when compared to pre-COVID-19, while chain hours were increased due to reduced activity. Overall, the COVID-19 crisis altered elephant management significantly, potentially affecting animal welfare resulting from changes in nutrition, health, exercise, and numbers of mahouts. We hope to use these data to develop better management plans and guidelines for elephant camps in Thailand so they can cope with the current and potential imminent pandemics that result in decreased tourism income. A follow-up study will measure health and welfare markers in relation to COVID-19 induced changes to determine if any camps adapted management to still meet elephant health and welfare needs, and could serve as models for responding to future pandemics.

## Introduction

The COVID-19 outbreak that began in 2019 is notable for its high rates of infection and fatalities, and enormous economic impacts worldwide (1), including those related to tourism (2, 3). It is estimated that global production output fell by 7% when only China went into lockdown, but reached 23% at the height of the crisis when they involved other nations (4). There are strong links between the strength of the tourist industry and economic growth within a country (5). However, because tourism is dependent on numbers of visitors, it is particularly vulnerable to disruptions caused by global pandemics (6). Thus, the COVID-19 pandemic has resulted in serious and widespread negative economic impacts on the economy of countries that depend on tourism income (2, 7), especially in regions with limited resilience to pandemic losses (8).

Although there have been some positive effects of the pandemic, such as reductions in greenhouse gases and air pollution (9, 10), overall, it has had adverse effects on wildlife tourism, both for businesses and animals, *in situ* and *ex situ*, leaving it in a more vulnerable position than before COVID-19. Venues involving animals (viewing or interactions) have been particularly hard hit (11–14). Due to reduced or no income, some zoos and wildlife rescue centers closed (12), with legitimate concerns over how shortages of food and staff will impact animal welfare (11). Likewise, a reduction in wildlife tourism experiences *in situ*, such as visiting national parks, protected areas, sanctuaries, has had negative impacts on tourist hotels, travel agencies, guides, and associated local communities (15), as well as conservation efforts because tourism funds a number of projects that protect habitats and the wildlife therein (16). Some free-ranging wildlife are reportedly going hungry because a popular tourist activity is feeding; for example, sika deer in Japan (17) and rhesus monkeys in Thailand (17, 18), although in one report, free-ranging elephants in Sri Lanka returned to wild foraging after a lockdown curtailed food handouts from tourists (19).

Thailand is the epicenter of elephant tourism and visiting an elephant camp is one of the most popular activities according to the Tourist Authority of Thailand. Elephants are the national symbol of Thailand and an integral part of Thai and Buddhist culture. There are ~3,500 captive elephants in Thailand, mostly (95%) privately owned (20, 21) and used primarily for tourism; thus they are also important to national economics. Most captive elephants in Thailand are in the north and northeast part of the country (~60%), primarily in Chiang Mai province (22). A recent survey of 33 elephant camps differing in size and years of operation in the region (23) found tourist activities varied and included hands-off opportunities like observation from afar, to feeding, bathing, and walking alongside, and to more interactive activities like riding with a saddle or bareback, and elephant shows. The question has always been – how do these tourist activities affect elephant health and welfare? Thus, a further evaluation of 122 elephants from 15 elephant camps using physical assessments of body condition, foot, and wound scores found that high energy foods (banana and sugar cane) were associated with obesity and alterations in total cholesterol (TC), low density lipoproteins (LDL), high density lipoproteins (HDL), triglycerides (TG), insulin, glucose, fructosamine and the ratio of glucose to insulin, while fecal glucocorticoid metabolite (fGCM) concentrations were lower in riding elephants, perhaps related to more exercise and better body condition (24, 25). However, poor foot scores were associated with longer work hours and walking distances and being on concrete, while skin wounds were related to improper restrain equipment used by mahouts (e.g., ankus or bullhook, chains) (26). Thus, while some tourist activities may benefit elephant health (24), others can contribute to poor welfare through long work hours, misuse of the ankus, stress associated with being too close to tourists, and harsh training to allow hands-on interactions (26, 27).

When the COVID-19 pandemic hit, the tourism landscape changed drastically. Upon recognition of the virus in March 2020, the Thai government banned all international travel (28), severely reducing foreign tourism and associated income. Consequently, tourist camps closed in Thailand, leading to further concerns over welfare of the elephants and mahouts. Therefore, the goal of this study was to document how elephant management changed a result of the international travel ban due to COVID-19. Surveys were conducted throughout the first 2 years of the country-wide lockdown, with data compared to before COVID-19 [(24, 25, 27), this study]. This information will then be used in subsequent multivariable studies to assess how management changes affected physiological function. It also will be used to devise plans for dealing with future pandemic-induced losses of income and identify areas that camps need to improve upon to adapt to inevitable future pandemics.

## Materials and methods

### Human ethical consent

This study was approved by the Faculty of Veterinary Medicine, Chiang Mai University Research Ethics Committee (HS1/2564).

### Animal ethical consent

This study was approved by the Institutional Animal Care and Use Committee, Faculty of Veterinary Medicine, Chiang Mai University, Chiang Mai, Thailand (FVM-ACUC, permit number S4/2564).

### Data collection

**Figure 1:**
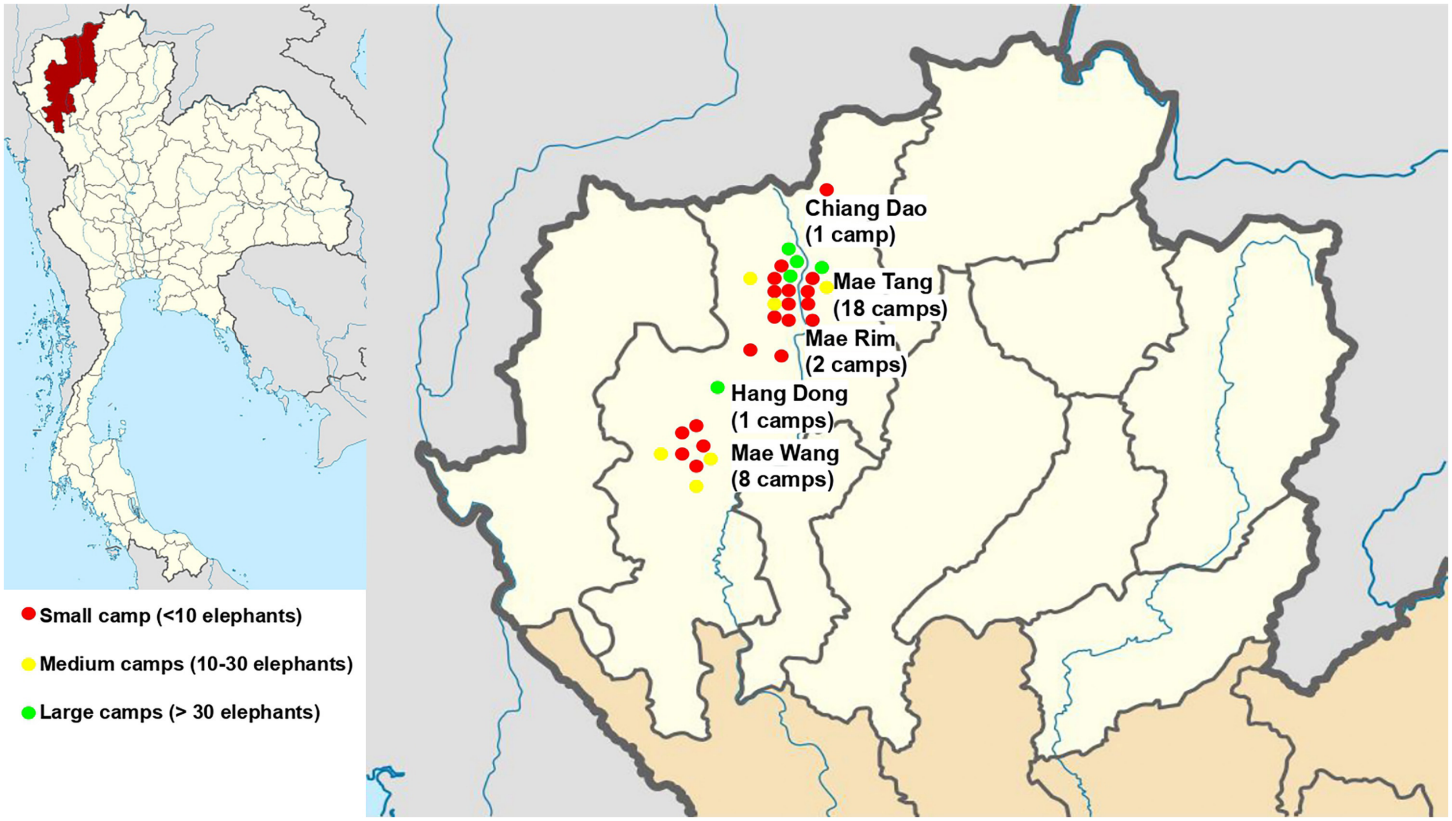
Distribution of elephant camps in this study. Colored dots represent the size of elephant camps based on numbers of elephants.

**Figure 2:**
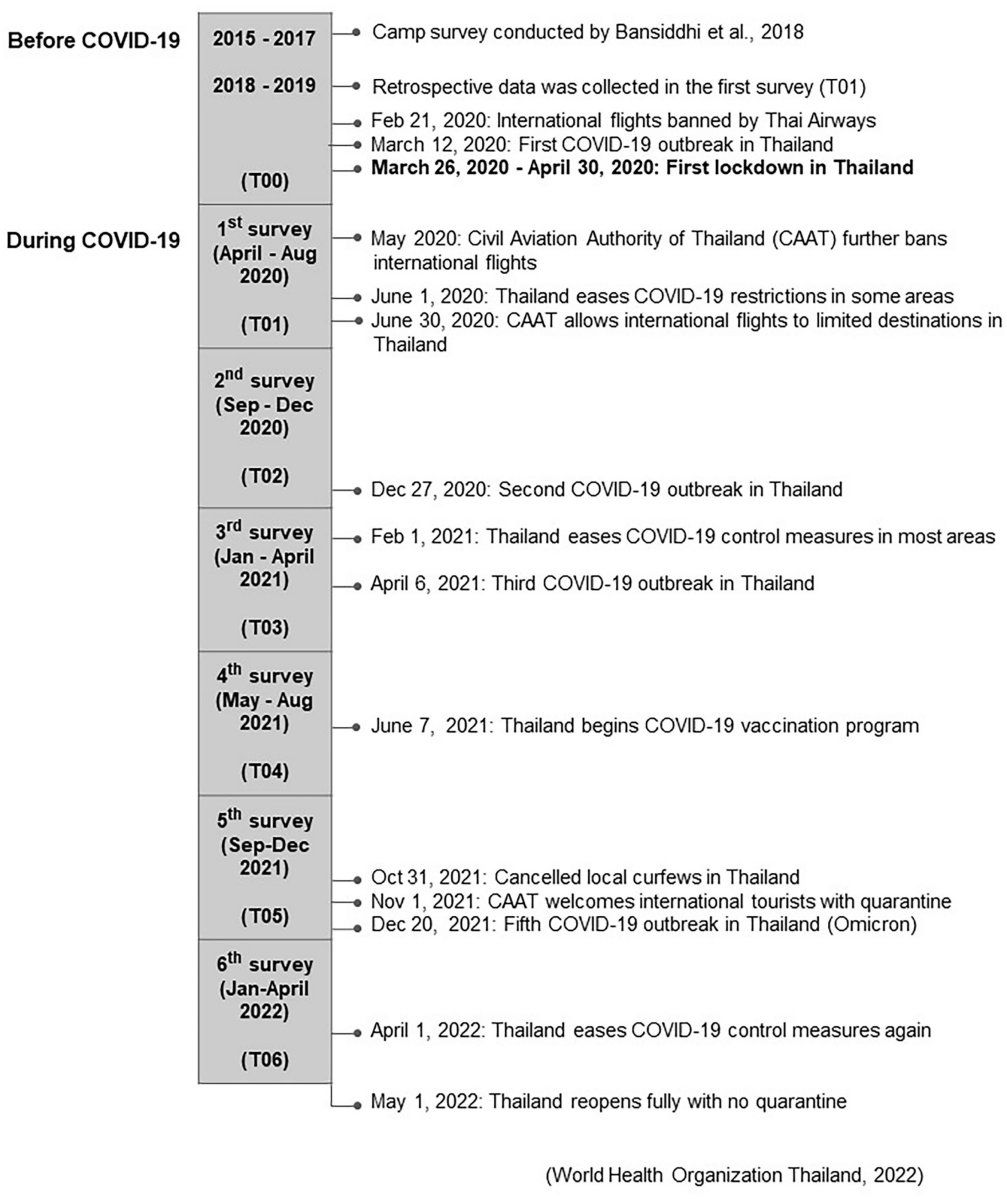
Associated events before the COVID-19 pandemic (T00), and across the six survey periods (T01-T06) during the study.

Data collection was carried out from April 2020 to April 2022. A total of 30 camps in five districts in Chiang Mai province were surveyed: Chiang Dao (one camp), Mae Tang (18 camps), Mae Rim (two camps), Hang Dong (one camp) and Mae Wang (eight camps) (Figure 1). These camps housed 495 elephants: 119 males (18.37 ± 1.67, range 3 months to 57 years of age) and 376 females (27.54 ± 0.94, range 8 months to 70 years of age), at the beginning of the study. Of these camps, 56% (*n* = 17) were considered small (<10 elephants), 27% (*n* = 8) were medium (10–30 elephants), and 17% (*n* = 5) were large (>30 elephants). Camps had been in operation for 0-5 (40%, *n* = 12), 6–15 (30%, *n* = 9) or >16 (23%, *n* = 7) years as of April 2020. The study consisted of questionnaire interviews with camp owners, managers, and/or camp veterinarians, and direct observations at elephant camps (Supplementary Table 1). Interviewers and observers were veterinarians experienced in working with elephants from the Veterinary Faculty at Chiang Mai University. The questionnaire consisted of questions that took approximately 60–90 min to complete: (1) camp management including sanitation, years of operation, elephant numbers, staff numbers, location, number of tourists, rest areas; (2) elephant management including tourist activities, chaining, restraint, access to drinking water, and musth management and nutrition; (3) mahout responsibilities, salaries and attitudes; and (4) health care consisting of sanitation practices, deworming program, veterinary care, and external sponsorship and funding support. Questions about camp management before COVID-19 were included in the first survey to capture data on operations in 2019 (Supplementary Table 1). Additional information on camp management and elephant activities before the COVID-19 pandemic also was available from Bansiddhi et al. (23). Follow-up surveys were then conducted every 4 months through April 2022 for a total of 2 years during the lockdown and international tourism ban (Figure 2).

### Statistical analysis

Descriptive statistics are reported as a percentage and the mean ± standard error of the mean (SEM). Statistical analyses were conducted using R program (version 3.4.0). Repeated survey data were analyzed using Generalized Estimating Equations (GEE) to determine how camp management variables changed over time (T00–T06). Differences in mean camp management variables (elephant numbers, staff numbers, mahout number, number of visitors, chain hours, frequency of access to drinking water, amount of roughage food, amount of high calories treats, and mahout salary) between times during COVID-19 were analyzed using by Dunnett's test using a *P-*value correction. Statistical significance was set at P < 0.05.

## Results

The timeline for associated events before the COVID-19 pandemic (T00), and across the six survey periods (T01–T06) during the study is shown in Figure 2. Thailand did not fully open to international travelers with no restrictions until 1 month after the last survey.

### Visitor, elephant, mahout, and staff numbers

The international travel ban initiated by the Thai government in April 2020 was followed by an immediate reduction in the number of visitors in T01 (Table 1, Supplementary Figure 2A), with no tourists visiting 60% of the camps (*n* = 17) and <1% of original tourist numbers in the rest, all of those being local Thais only. Tourist numbers remained low even as some restrictions were lifted in mid-2020, when international travel was allowed, but with limitations (quarantine for 14 days and only in some locations) (Figure 2). In the last two surveys, visitor numbers had begun to increase again, but were still only 7% of pre-COVID numbers (Table 1, Supplementary Figure 2A).

**Table 1:** Summary of parameters (mean ± SEM, range) related to management of elephants in tourist camps in Chiang Mai province, Thailand, in each period from surveys conducted over 2 years during the COVID-19 pandemic compared to the year pre-COVID-19.

**Parameters**	**T00** **before** **COVID-19^1^**	**Time periods during COVID-19**					
		**T01** **(April 2020–** **August 2020)**	**T02** **(September 2020–** **December 2020)**	**T03** **(January 2021–** **April 2021)**	**T04** **(May 2021–** **August 2021)**	**T05** **(September 2021–** **December 2021)**	**T06** **(January 2022–** **April 2022)**
**Visitors/day**	99.82 ± 30.00^a^	1.74 ± 0.67^b^	2.18 ± 0.87^b^	2.18 ± 0.87^b^	1.82 ± 0.87^b^	4.21 ± 1.14^b^	7.39 ± 1.12^b^
	8–600	0–15	0–20	0–20	0–20	0–30	0–30
**Number of elephants**	16.50 ± 3.62^a^	14.66 ± 3.45^b^	14.34 ± 3.41^b^	13.38 ± 3.28^b^	12.97 ± 3.23^b^	12.63 ± 3.13^b^	11.83 ± 2.86^b^
	2–69	1–67	0–65	0–63	0–59	0–55	0–55
**Number of mahouts**	16.37 ± 3.71^a^	9.00 ± 1.88^b^	8.89 ± 2.07^b^	8.07 ± 1.87^b^	8.00 ± 1.88^b^	7.50 ± 1.67^b^	6.79 ± 1.40^b^
	2–66	0–40	1–40	1–39	1–39	1–32	1–30
**Mahout/** **elephant ratio**	0.99^a^ 1:1	0.64^b^ 1:2	0.58^b^ 1:2	0.56^b^ 1:2	0.56^b^ 1:2	0.55^b^ 1:2	0.54^b^ 1:2
**Number of Staff**	30.5 ± 7.97^a^ 4–209	14.90 ± 3.53^b^ 3–80	14.97 ± 3.52^b^ 3–80	11.55 ± 2.59^b^ 2–60	11.55 ±2.59^b^ 2–60	11.17 ± 2.38^b^ 2–50	9.59 ± 2.16^b^ 2–45
**Walk distance (km/day)**	4.12 ± 0.70^a^	1.28 ± 0.15^b^	1.04 ± 0.16^b^	0.76 ± 0.11^b^	0.85 ± 0.10^b^	1.29 ± 0.12^a^	1.54 ± 0.15^a^
	0.6–20	0.3–4	0.5–3	0.3–3	0.3–3	0.5–3	0.5–3
**Access to water/day** ^2^	3.33 ± 0.12^a^	2.90 ± 0.07^b^	2.00 ± 0.12^b^	1.27 ± 0.10^b^	1.23 ± 0.09^b^	1.23 ± 0.09^b^	1.23 ± 0.09^b^
	2–4	1–3	1–3	1–3	1–3	1–3	1–3
**Chain time (hours)** ^2^	15.85 ± 0.42^a^	18.97 ± 0.63^b^	21.47 ± 1.24^b^	23.96 ± 1.52^b^	25.75 ± 1.72^b^	23.75 ± 1.53^b^	21.16 ± 1.06^b^
	0–19	0–24	0–48	0–48	0–48	0–48	0–48
**Chain length (m)**	3.85 ± 0.47^a^ 0–12	5.47 ± 0.78^b^ 0–15	5.45 ± 0.79^b^ 0–15	5.31 ± 0.81^b^ 0–15	5.35 ± 0.80^b^ 0–15	5.09 ± 0.79^b^ 0–15	5.09 ± 0.79^b^ 0–15
**Roughage (kg/day)**	213.45 ± 14.07^a^ 100–400	208.3 ± 14.10^b^ 100–400	173.45 ± 10.32^b^ 80–300	164.29 ± 8.02^b^ 90–250	148.21 ± 6.28^b^ 90–200	147.86 ± 6.01^b^ 90–200	152.50 ± 6.62^b^ 90–250
**Supplements (kg/day)**	26.0 ± 1.82^a^	19.0 ± 1.32^b^	10.5 ± 0.94^b^	6.5 ± 0.61^b^	6.3 ± 0.55^b^	6.3 ± 0.55^b^	9.6 ± 0.51^b^
	10–50	10–30	2–25	1–15	5–15	5–15	5–15

1 Based on interview questions included in T01 survey about conditions in 2019.

2 Some elephants were chained for more than 24 hours at a time, so these data represent contiguous hours in any one time period.

a, b Different letters in the same row indicate significant statistical differences compared each time period to before COVID (T00) when subjected to Dunnett's Multiple Comparison (P < 0.001).

Elephant numbers at each camp were decreased by about 11% soon after camps closed to 39% at the end of the survey period (Table 1, Supplementary Figure 2B). At some camps, mahouts returned elephants to their home village (56.7%, *n* = 17), while some were sold to other camps (30%, *n* = 9). Three mahouts (10%) took elephants to log in Surin province, while two owners allowed elephants to stay at a temple (6.7%, *n* = 2) (Table 1, Supplementary Figure 2C). There was a 45% decrease in mahout numbers almost immediately that then stabilized through 2021 (Table 1, Supplementary Figure 3C) dropping to a low of 59% of pre-COVID numbers in T06. Overall, the decrease in numbers of elephants was less than the reduction in numbers of mahouts so the overall ratio of mahouts to elephants dropped from around 1:1 at T00 to 1:2 throughout T01–T06 (Table 1). A 50% reduction in other staff, including gardeners, drivers, cleaners, cooks, and guides also was observed across facilities shortly after the lockdown in T01 (Table 1, Supplementary Figure 2D), with the lowest percentage (29%) observed at the end of the study.

### Work activities

**Figure 3:**
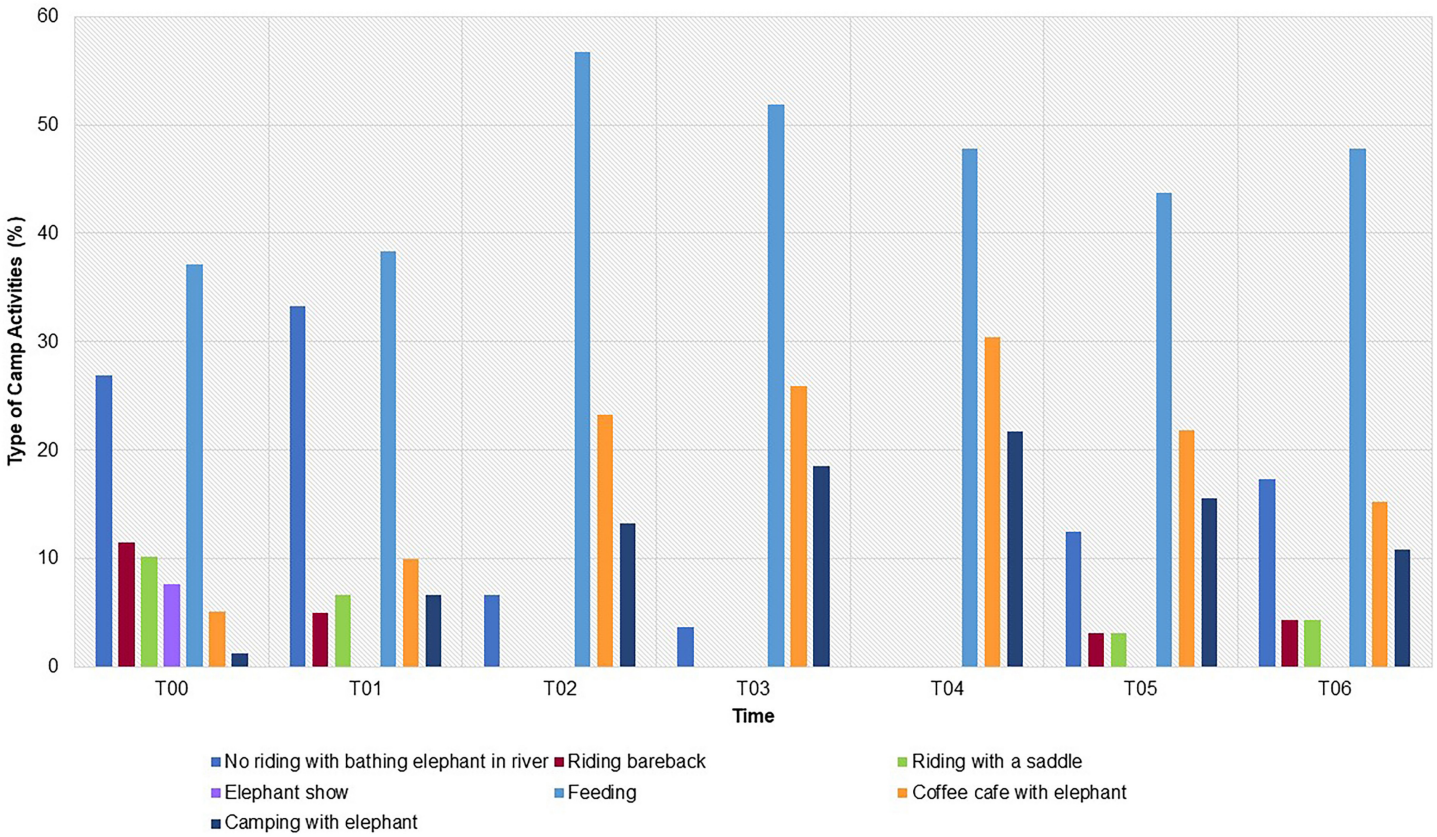
Changes in tourist activities at elephant camps in northern Thailand before (T00) and through six survey periods [T01 (April–August 2020), T02 (September–December, 2020), T3 (January–April, 2021), T04 (May-August 2021), T05 (September–December, 2021) and T06 (January–April, 2022)] during the COVID-19 pandemic. Data represent the percentage of camps engaged in each activity across time periods.

**Figure 4:**
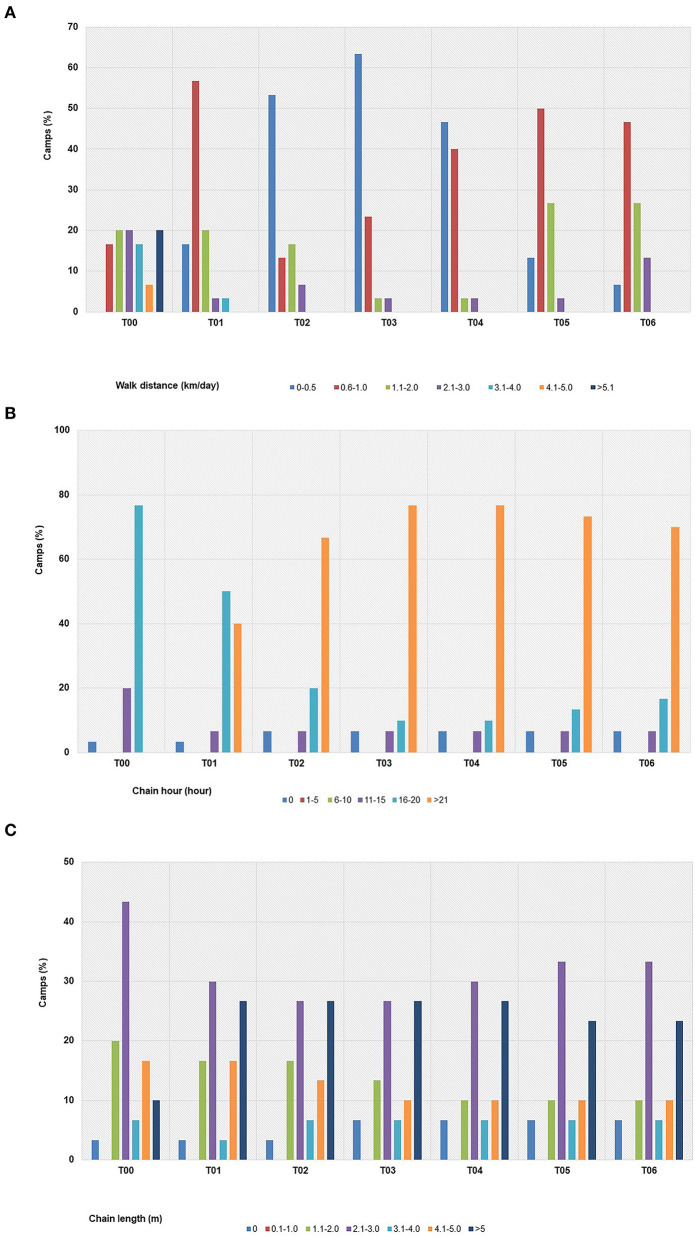
Changing trends of elephant exercise **(A)** walking distance, **(B)** chain hours, and **(C)** chain length at individual elephant tourist camps in northern Thailand before (T00) and through six survey periods [T01 (April–August, 2020), T02 (September–December, 2020), T3 (January–April, 2021), T04 (May–August, 2021), T05 (September–December, 2021) and T06 (January–April, 2022)] during the COVID-19 pandemic.

Pre-COVID-19 information collected as part of the initial T01 survey (designated T00) found the main tourist activities were no-riding and bathing (27% of camps) followed by feeding (37%), and then bareback (12%) or saddle (10%) riding, and shows (8%) (Figure 3, Supplementary Figure 1). Additional activities not described in prior studies included coffee café with elephants (5%), where a group of tourists interact with elephants by feeding bananas or sugar cane and/or observation from the coffee bar, and camping with elephants (1%), where tourists stay overnight in a tent with feeding and observation opportunities. With no tourists, elephant activities in the majority of camps ceased and so there was little if any exercise in the form of riding, foraging, or other work (Figure 3). At the beginning of study (T00), elephants walked on average over 4 km/day part of tourist activities, with some walking up to 20 km/day (Table 1, Figure 4A). After the lockdown, daily walking distances at most camps (70%, *n* = 21) were reduced, with a low in T03 (< 0.8 km/day). As shown in Figure 4A, no camps walked elephants less than 0.6 km/day before COVID-19, while no elephants were exercised more than 4 km/day after camps closed in T01. Riding activities declined to less than 10% in T01 and were halted altogether through T05, when a small number of local tourists (<5% of camps) returned for these activities (Figure 3). At the end of the study, the percentage of camps providing at least some walking opportunities was 47% (*n* = 14) (Figure 3). By contrast, 46% of camps (*n* = 19) continued to allow local tourists to feed supplements purchased for elephants, like bananas and sugar cane, throughout the study period. Other activities that appealed to local Thai people increased, such as coffee café and elephant camping, which made up a greater percentage of activities involving elephants as the pandemic progressed, in addition to feeding (Figure 3).

Before COVID-19, mahouts bathed elephants in a river (53% of camps), often with tourists, or by spraying with a hose (66%) at a frequency of two (23%) to four (30%) times per day Table 1, Figure 3). When camps closed, bathing frequency was reduced to 1–3 times per day, but over time, fewer camps were doing it. By T03, bathing times were less than half those in T01, and many camps (80%) stopped bathing altogether. Whereas before COVID-19, one mahout would bathe one elephant, at the end of the study a mahout might bathe a group of elephants, and at a decreased frequency (Table 1).

### Chaining, housing, rest areas

With the reduction in work activities, there was an increase in chaining time at 77% of the camps (*n* = 23) (Table 1, Figure 4B). Chaining time already averaged 16 hours/day before the lockdown, although there was considerable variability across camps, ranging from 0 to 19 h in T00 and 0–48 h in T06 (Table 1, Figure 4B). By T02, some camps (7%, *n* = 2) started chaining elephants for 48 straight hours. Only four camps (13%) allowed elephants to roam freely without chaining, and that stayed constant throughout the study (Table 1, Figure 4B). No camps chained elephants for >21 h before COVID-19, but after T03, most did (Table 1, Figure 4B).

At the beginning of the study, chain lengths at most camps were 2.1–3.0 meters (T00), with only 10% using chains >5 m (Table 1, Figure 4C). After the lockdown (T01), 23–27% of camps increased the length of the chains used. By contrast, chain lengths were shortened at three camps because of limited and more restricted space (Table 1, Figure 4C). During COVID-19, most camps (67%, *n* = 20) chained elephants under a covered shed, while some (33%, *n* = 10) kept animals in sheds and/or woodlands. In three camps, elephants were allowed to roam free in neighboring forests while being restrained by heavy chains, while at two, they were allowed to roam freely around the camp.

### Nutrition

**Figure 5:**
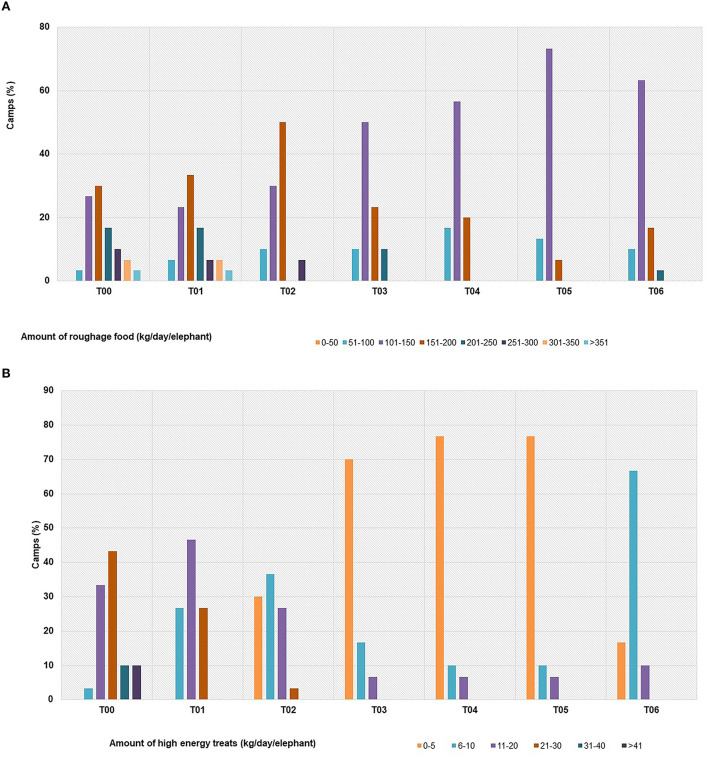
Changing trends of food provided **(A)** roughage **(B)** supplement at individual elephant tourist camps in northern Thailand before (T00) and through six survey periods [T01 (April–August, 2020), T02 (September–December, 2020), T3 (January-April, 2021), T04 (May-August 2021), T05 (September–December, 2021) and T06 (January-April, 2022)] during the COVID-19 pandemic. Types of high energy treats are described in Supplementary Figure 3B.

The types of roughage offered did not change significantly during COVID-19 (Supplementary Figure 3A), although the amounts fed were reduced over time, averaging only ~70–80% of those in T00 (Figure 5A and Table 1). The vast majority of camps fed napier grass, which continued throughout the study (Table 1, Supplementary Figure 3A); however, the number of camps feeding cornstalks declined from 67% in T00 to 47% from T01 onwards (Supplementary Figure 3A). In T04, over half of the camps tried feeding straw, but that was discontinued by the next survey (Supplementary Figure 3A). Elephants were fed a variety of supplements before COVID-19, most commonly bananas, sugar cane and tamarind (Table 1, Supplementary Figure 3B). These items continued to be offered through 2020, although fewer camps did so; 86% of camps fed sugar cane in T00 but only 40% did in T01, while tamarind went from 100% to less than 3% in just a few months (Figure 5B). Overall, the amount of supplemental, higher calorie food was reduced by 57% across camps (Figure 5B and Table 1), going from feeding 10–50 kg/day in T00 to 5–15 kg/day in T06. Beginning in T01 some supplements like bananas, sugar cane and other seasonal fruits like pumpkin, watermelon, cantaloupe, melon, and mango were donated by local Thai people.

### Health care

Before COVID-19, four camps had their own full-time elephant veterinarian on site, while other camps were visited twice a year by veterinarians from the National Elephant Institute (NEI) (*n* = 5), the Center of Elephant and Wildlife Health, Chiang Mai University (CMU) Animal Hospital (*n* = 9), the Department of Livestock Development (DLD), National Institute of Elephant Research and Health Service (*n* = 1), or the Thai Elephant Alliance Association (TEAA) (*n* = 3) that conducted routine health checks and provided deworming services. After the lockdown, only three camp veterinarians remained, and all at a reduced salary (20–30% of T00). Numbers of veterinarians working for the TECC, CMU, DLD and TEAA remained the same and they continued to visit camps for routine care, but took on additional tasks, such as foot care, gastrointestinal tract (GI) treatment, wound care and other health problems because of the reduction in mahouts and elephant exercise activity.

### Mahout management and mahout attitudes

Mahouts continued to care for all aspects of the elephants' daily lives, including walking, providing food and water, cleaning enclosures, and bathing (Supplementary Figures 1, 4), although amounts of time devoted to these activities often were reduced. A total of 87% (*n* = 214) of surveyed mahouts answered questions about attitudes toward management changes during COVID-19 (Table 2, Supplementary Figure 4). Mahout salaries were reportedly decreased by 60% during the pandemic, as were self-reported feelings of stress and sadness (Table 2), although by T06, those feelings had decreased somewhat. By contrast, fear of layoffs was reported by only by a third of mahouts soon after camps closed, but increased as the pandemic progressed to over 90% in T04–T05 (Supplementary Figure 2C). By the last survey, the percentage was still close to two-thirds. Some mahouts reported getting second jobs, such as a gardener or construction worker, depending on the camp.

**Table 2:** Mean (± SEM) and percentage of answers on the mahout surveys (*n* = 214) conducted over 2 years during the COVID-19 pandemic.

**Parameters**	**Time periods during COVID-19**					
	**T01** **(April 2020-** **August 2020)**	**T02** **(September 2020-** **December 2020)**	**T03** **(January 2021-** **April 2021)**	**T04** **(May 2021-** **August 2021)**	**T05** **(September 2021-** **December 2021)**	**T06** **(January 2022-** **April 2022)**
Mahout salaries (Baht Thai)^1^	4,900 ± 381^a^	4,736.84 ± 363^b^	4,070.95 ± 363^b^	4,070.95 ± 363^b^	4,070.95 ± 363^b^	4,070.95 ± 363^b^
	3,000–9,000	3,000–9,000	3,000–9,000	3,000-9,000	3,000–9,000	3,000–9,000
Mahout attitudes						
Feel stressed (%)	87.36^a^	81.9^b^	86.16^b^	83.33^b^	76.19^b^	63.16^b^
Feel sad (%)	78.78^a^	59.47^b^	67.24^b^	46.30^b^	38.10^b^	31.58^b^
Worried about layoffs (%)	33.62^a^	69.44^b^	78.37^b^	92.59^b^	90.48^b^	52.63^b^

1 Mahout salaries before COVID-19 averaged 10,048 ± 754 Baht Thai.

a, b Different superscript across rows indicate significant statistical differences compared each time period to T01 when subjected to Dunnett's Multiple Comparison (P < 0.001).

## Discussion

This study presents survey results on the effects of the COVID-19 pandemic and international travel ban on elephant tourist camp management in northern Thailand. The study population represented 61% of the total elephant numbers in the Chiang Mai region (14% overall in Thailand). Compared to pre-COVID-19, data revealed major changes in camp and elephant management occurred as a result of a loss in tourism income. Reductions in exercise opportunities, increases in chaining time, changes in diets, and loss of mahouts all were observed and fully expected to have significant impacts on animal wellbeing. In addition to surveys, biological samples and health data also were collected for future studies to measure physiological responses (i.e., body condition, stress, metabolic, liver, muscle function, and behavior), data that will be important to understanding how changes in diet, health care, and exercise affected aspects of individual elephant welfare.

### Visitor, elephant, mahout, staff numbers

In the present study, elephant numbers declined by more than 30% over time as mahouts returned to villages or elephants were sold, whereas as staff was reduced by 50% or more as the pandemic progressed. In Nepal, the captive elephant population also decreased by 18.5% during COVID-19 since an earlier report in 2012, in part related to illegal selling of privately owned elephants to Indian entrepreneurs (29). To our knowledge, there are no other studies documenting the effect of the COVID-19 pandemic on the management of elephants used primarily for tourism. However, it can be interfered that changes in camp management, including reducing the mahout to elephant ratio, will have significant effects on health and welfare, and cause stress in elephants forced to adapt to new environments (30).

### Work activities

Before COVID-19, elephants generally worked from 8.00–10.00 to 14.00–15.00 h depending on seasonal tourist activities, and were chained primarily during non-tourist hours (31). The types of elephant tourist activities identified in T00 (before COVID-19) were similar to those reported earlier and included riding with a saddle, riding bareback, no-riding, bathing, and shows (23). Before the pandemic, walking distances averaged 4 km/day, with some elephants walking up to 20 km/day during trekking. Those distances were comparable to earlier findings of approximately 5–10 km/day in North American (32), Melbourne (33) and Dublin (34) zoos, tourist camps in Thailand (23), forest camps in India (35), and estimates for wild elephants (36–38). These were drastically reduced within months of the lockdown and remained low throughout the study period. However, there were four camps that made an effort to take elephants for walks, albeit at a reduced frequency. This is concerning because a previous study in North American zoos showed elephants that walked 14 h or more per week were at a reduced risk of being obese (39), a problem identified in Thailand that was ameliorated by exercise (e.g., riding) (24, 25, 40). However, it is important to point out that although riding and other activities can be good for general body condition and metabolic health (24, 25), the amounts and types of work, and training needed for elephants to participate in interactive tourist activities can have numerous negative consequences (27).

In northern Thailand, the process of Phajaan was originally designed to break an elephant's spirit so it could be handled more easily, and generally included restraining in a small enclosure with chains and harnesses to limit movement, hitting with an ankus, and then rewarding with bananas over a period of 5–10 days (31, 41). Today, Phajaan is mostly ceremonial with blessings conducted to prevent bad spirits from harming the calf. Some camps train their own baby elephants, while others send them to the National Elephant Institute (NEI), where more positive methods are now being used and based on training provided by western experts (41). In the livestock industry, Grandin (42) noted that working with large animals carries some inherent risks, and that training animals to cooperate with handling techniques can lessen anxiety and accidents. While more camps report using positive training techniques today, most elephants are still controlled with an ankus (i.e., bullhook; 85% of camps) (23), which if used improperly can injure elephants (31). For example, 27% of elephants controlled by an ankus had associated wounds, and higher wound scores were associated with higher fGCM concentrations (26, 27). Ill-fitting saddles or inadequate or inappropriate padding material also can cause lesions (43), and although not properly studied, the shape of the backbone is believed to play a role, with higher ridgelines being more prone to saddle injuries. Following this study, improvements in saddles and padding were made (43), resulting in fewer lesions (5%) in a subsequent survey (26), while another study showed carrying loads up to 15% of the elephants' body weight did not alter gait dynamics (44).

A small percentage of camps (~8%) put on elephant shows, which have their own welfare concerns. Hernias, arthritis, lameness, and joint issues may be caused by repeated abnormal positions during performances, as has been shown in circus elephants (45). These shows were curtailed soon after the lockdown in T01. Finally, it is not always clear how or if camps are addressing the mental health needs of elephants, particularly in relation to socialization (21, 27). However, one positive sign from a 2018 survey is that newer camps appear to be providing more opportunities for elephants to be together, to socialize and play, especially during bath time (23). Positive social connections between animals, even those that are not related, can operate as a calming force against difficult situations and improve general health and wellness (46–48). However, any progress in this area was curtailed during the COVID-19 lockdown, when most elephants were chained for prolonged periods of time with no ability to socially interact.

### Chaining, housing, rest areas

Chaining is a way to restrict movement of elephants at facilities with limited space or no other means of containing them. The vast majority of camps in northern Thailand use chains to control elephants, especially at night; only a few have enclosures to allow elephants untethered movements (23). Even before the pandemic, elephants in this study were chained on average nearly 16 h/day. That increased to up to 48 contiguous hours at some camps. Chaining for extended amounts of time to restrict movement can cause problems with joints and feet (49, 50) and be a source of psychological stress. In a recent survey of 283 elephants at 20 elephant camps in Chiang Mai province conducted the lockdown, 57% exhibited stereotypic behavior (51), an indicator of poor welfare. Swaying was the most common, followed by weaving and pacing, and was more common in younger elephants. Previous research has demonstrated a strong positive association between chaining and the degree of stereotypic behavior compared to elephants kept in an enclosed space that allows some free movement (52, 53). The Food and Agriculture Organization of the United Nations published a Elephant Care Manual for Mahouts and Camp Managers a decade ago that states that chains to confine adult elephants in Asia should be 20–30 m in length (54), which is rarely adhered to in Thailand; chains typically average 3 m during the day and 6 m at night (23). In southern India, a higher prevalence of stereotypies were observed in elephants chained for 20 and 18 h/day in Hindu temples (49%) and private camps (25%), respectively, compared to those chained by the Forest Department for only 6 h/day (7%) (55). In western zoos, chaining is acceptable during medical treatments or other short-term interventions, but not for prolonged restraint. In the current study, average chain length was only 2–3 m at beginning of the study, but was increased to more than 5 m after T05 potentially to help mitigate the reduction in activity levels, but also because the density of elephants under a shelter was also lower. Western zoos require elephants have access to both indoor and outdoor spaces (56) and for the most part, elephants in Thailand were kept in covered sheds or forest canopies (23).

### Nutrition

Few camps in Thailand are located in forested areas that allow elephants to forage naturally, and even those that are still have to supplement because of degraded habitats, especially during the dry season (57). In general, elephants consume about 5% of their body weight on a wet weight basis, depending on sex and age; thus, an elephant cow needs 150–175 kg/day while bulls require 200–275 kg/day (58). Before COVID-19, elephants were fed roughage before morning work activities at 6.00–8.00 h and again at 17.00–21.00 h in the evening (23), and that was still the case during the pandemic. However, while the average amount of roughage offered was similar to other studies in T00, it was reduced from 200 kg/day to 150 kg/day during the pandemic. At most camps, tourists often pay to feed elephants a number of supplementary foods, such as banana and sugar cane and other seasonal fruits, which often reach 30 kg/day during the high tourist season (40). That was similar to the ~26 kg/day amount fed pre-COVID-19, but was reduced significantly to a low of 6.3 kg/day in T04, and provided mostly by local Thai tourists. Although not quantified, a reduction in foraging at some camps was an indirect consequence of the lack of tourists, and also reduced numbers of mahouts, taking them for walks in the forest. One question to be addressed in follow up studies is how changes in diet affected body condition and metabolic activity, and whether more limited feeding of high calorie treats might benefit elephant health and reduce the incidence of obesity, or would those improvements be offset by concomitant reductions in physical activity.

### Health care

Although the number of elephant veterinarians did not change significantly during the COVID-19 pandemic (only one camp veterinarian was let go), salaries were reduced and attitudes were negatively affected. There also was an increase in reported elephant health problems during the shutdown between 2019 and 2022 (59), presumably due to reduced care with fewer mahouts being available to do daily health checks. Likewise, more incidences of colic could have been related to reductions in exercise and associated impaired GI movement, in addition to poorer quality roughage.

As the pandemic progressed and camp incomes were reduced, veterinarians and veterinary assistants were increasingly supported by outside organizations, including Asian Elephant Support, Southern Thailand Elephant Foundation through the Thai Elephant Alliance, the Thai Elephant Federation, GTAEF Helping Elephants Foundation, and the Elephant Care International Healthcare and Welfare Lifeline Fund. In addition, there was some government assistance from the Tourism Authority of Thailand (TAT) to help elephant communities, and low-interest loans were provided by the Ministry of Finance for elephant camp operators.

### Mahout management and attitude

Many years of research in the livestock industry have highlighted the significance of good human-animal relationships (HARs) on animal welfare and productivity, leading to recommendations for stockpersons to undergo cognitive-behavioral training as well as the inclusion of HAR assessments in on-farm welfare audits (60–62). Mahouts play an important role in the life of elephants, both positive and negative. They can engender fear as in punishment for misbehavior or gradually strengthen and foster compassionate relationships (31). Mahouts and elephants often develop special bonds that are rarely found in other human-animal interactions, and can have positive impacts on health and welfare (63, 64). Ultimately, the wellbeing of elephants is inextricably tied to the experience and compassion of mahouts, which unfortunately appears to be dwindling across Asia (63, 65). Strong ties between mahouts and elephants also can predict levels of cooperation. When elephants were asked to cross a novel surface (low bridge), those that had worked with their handler for over a year were more willing to cross it than those with a shorter relationship (66). Likewise, elephants responded more, and faster, in behavioral tasks in response to mahouts they had known longer (63). In zoo elephants, positive keeper attitudes were related to lower mean serum cortisol concentrations as a measure of stress, while keeper work satisfaction was predicted by the strength of keeper-elephant connections (67).

Given mahout welfare is a critical component of elephant welfare, the mental health and physical fitness of mahouts is so important (68). The COVID-19 pandemic dramatically affected mahouts, not just in terms of salary but overall attitudes and quality of life (35, 68). As mahout salaries were reduced, feelings of stress and sadness increased. In particular was an increased concern over layoffs as the pandemic proceeded. Thus, it was clear that plans to deal with future pandemics must include ways to support mahouts as the centerpiece of elephant care and welfare.

## Conclusion

This study found the COVID-19 pandemic had direct and significant effects on elephant camp management as a result of a loss in tourist income. Reductions in exercise opportunities and food provided, increases in chaining time, and fewer mahouts were observed, which could have significant impacts on elephant welfare. The next step will be to correlate measures of body condition, fGCM concentrations, metabolic and muscle function biomarkers, lipid panels, and behavior to determine how these management changes affected the health and welfare of specific elephants. It will also be key to identify any camps that adapted management in a way that still met elephant health and welfare needs, and which could serve as models for responding to future pandemics.

There were several notable findings from the responses to this pandemic. One was that most elephants in Thailand are located in areas with limited access to natural habitats for foraging. Before the pandemic, this problem was mitigated by large numbers of tourists providing an income to camps to purchase roughage, and by buying treats to feed elephants directly. In addition, at many camps, elephant care was based on daily tourist activities (feeding, walking, trekking, etc.) rather than allowing elephants to roam free to forage and socialize as a means of exercise. Therefore, when guests were not around, elephants were simply chained. To plan for future pandemics, while it is not possible for all camps in Chiang Mai at the present time, it is strongly recommended that they be established near forests to provide adequate space for elephants to roam and forage regardless of whether tourists are around or not. However, resistance by government or community agencies to allowing elephants access to forested areas for fear habitat would be destroyed in the long-term, is an impediment. Some camps have planted grass fields and grow their own food, a solution that could be expanded to other facilities. Those actions could reduce the food budget, while foraging would serve as natural enrichment. Another recommendation is to limit elephant numbers according to the space available at each camp and adjoining land. Keeping elephant numbers in proportion to the space could allow management to provide longer chains (20–30 m) providing more freedom of movement. Thus, we suggest it is important to manage appropriate numbers of elephants suitable for the natural environment, with responsible mahouts to care for them by encouraging daily exercise and good quality food. Opportunities to socialize with compatible elephants should be provided, even in restricted areas. These adaptations could ensure better welfare for elephants, not just during this pandemic, but going forward once tourism returns to pre-pandemic levels, and in anticipation of future crises.

## Data availability statement

The original contributions presented in the study are included in the article/Supplementary material, further inquiries can be directed to the corresponding authors.

## Ethics statement

The studies involving human participants were reviewed and approved by Faculty of Veterinary Medicine, Chiang Mai University Research Ethics Committee (HS1/2564). The patients/participants provided their written informed consent to participate in this study. The animal study was reviewed and approved by the Institutional Animal Care and Use Committee, Faculty of Veterinary Medicine, Chiang Mai University, Chiang Mai, Thailand (FVM-ACUC, permit number S4/2564). Written informed consent was obtained from the owners for the participation of their animals in this study. Written informed consent was obtained from the individual(s) for the publication of any potentially identifiable images or data included in this article.

## Author contributions

JS conceived and designed the experiments, performed the experiments, analyzed the data, contributed reagents/materials/analysis tools, prepared figures and/or tables, authored, reviewed drafts of the paper, and approved the final draft. JB conceived and designed the experiments, contributed reagents/materials/analysis tools, funding acquisition, reviewed drafts of the paper, and approved the final draft. PB conceived and designed the experiments, performed the experiments, contributed reagents/materials/analysis tools, and approved the final draft. CT performed the experiments, analyzed the data, contributed reagents/materials/analysis tools, funding acquisition, and approved the final draft. VP analyzed the data, contributed reagents/materials/analysis tools, prepared figures and/or tables, and approved the final draft. JK conceived and designed the experiments, performed the experiments, analyzed the data, prepared figures and/or tables, contributed reagents/materials/analysis tools, funding acquisition, project administration, authored, reviewed drafts of the paper, and approved the final draft. All authors contributed to the article and approved the submitted version.

## Funding

This study was supported in part by the Smithsonian Conservation Biology Institute (SCBI, USA) through a grant from the Shared Earth Foundation as part of a Memorandum of Understanding with Faculty of Veterinary Medicine, Chiang Mai University (grant number R000032244) and the Center of Elephant and Wildlife Health, Chiang Mai University Animal Hospital, Chiang Mai, Thailand (grant number 59/2565).

## Conflict of interest

The authors declare that the research was conducted in the absence of any commercial or financial relationships that could be construed as a potential conflict of interest.

## Publisher's note

All claims expressed in this article are solely those of the authors and do not necessarily represent those of their affiliated organizations, or those of the publisher, the editors and the reviewers. Any product that may be evaluated in this article, or claim that may be made by its manufacturer, is not guaranteed or endorsed by the publisher.

## Supplementary material

Supplementary Figure 1 Examples of camp management and mahout routine work during COVID-19. **(A)** Elephant in a nearby forest, **(B)** Walking activity, **(C)** Elephant at coffee café, **(D)** Covered shed with elephants chained near each other **(E)** Bathing elephant by mahout **(F)** Supplement food from private donations. Photography by Jarawee Supanta.

Supplementary Figure 2 Changes in the number of visitors **(A)**, elephants **(B)**, mahouts **(C)** and other staff **(D)** at individual elephant tourist camps in northern Thailand before (T00) and through six survey periods [T01 (April–August, 2020), T02 (September–December, 2020), T3 (January–April, 2021), T04 (May–August, 2021), T05 (September–December, 2021) and T06 (January–April, 2022)] during the COVID-19 pandemic and corresponding reduction in tourist numbers.

Supplementary Figure 3 Variety of roughage **(A)** and supplement **(B)** foods offered to elephants at camps in northern Thailand before (T00) and through six survey periods [T01 (April–August 2020), T02 (September–December, 2020), T3 (January–April, 2021), T04 (May–August, 2021), T05 (September–December, 2021) and T06 (January–April, 2022)] during the COVID-19 pandemic. Data represent the percentage of camps offering each food type across time periods.

Supplementary Figure 4 Mahout activities of the daily routine at individual elephant tourist camps in northern Thailand through six survey periods [T01 (April–August, 2020), T02 (September–December, 2020), T3 (January–April, 2021), T04 (May–August, 2021), T05 (September–December, 2021) and T06 (January–April, 2022)] during the COVID-19 pandemic.

Supplementary Table 1 Questionnaire of Project: An assessment of the elephant camp management in the COVID-19 crisis for better health on elephant welfare in Chiang Mai tourist industry. The full questionnaire sheet used to record information during camp visits.

Supplementary Data The raw data.
