# Supplementary material for: Effect of the COVID-19 pandemic and international travel ban on elephant tourist camp management in northern Thailand
Source: Front Vet Sci. 2022 Dec 2;9:1038855. doi: 10.3389/fvets.2022.1038855 (PMC9755861; doi:10.3389/fvets.2022.1038855)
Supplement: Supplementary Figure 1 — Examples of camp management and mahout routine work during COVID-19. (A) Elephant in a nearby forest, (B) Walking activity, (C) Elephant at coffee café, (D) Covered shed with elephants chained near each other (E) Bathing elephant by mahout (F) Supplement food from private donations. Photography by Jarawee Supanta. [file Data_Sheet_1.docx]

Supplementary Material

# Supplementary Data

- The raw data

# Supplementary Figures and Tables

## Supplementary figures

**A.**
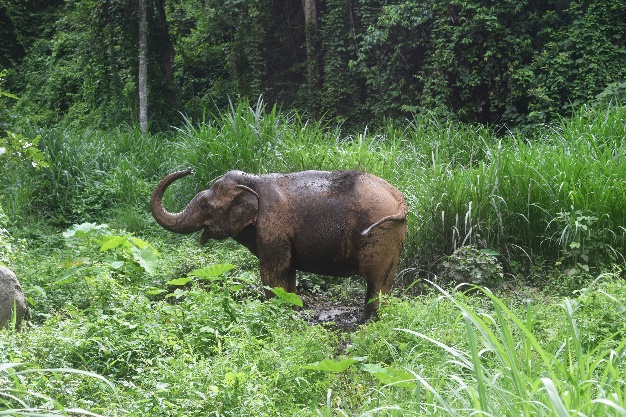
 **B.**
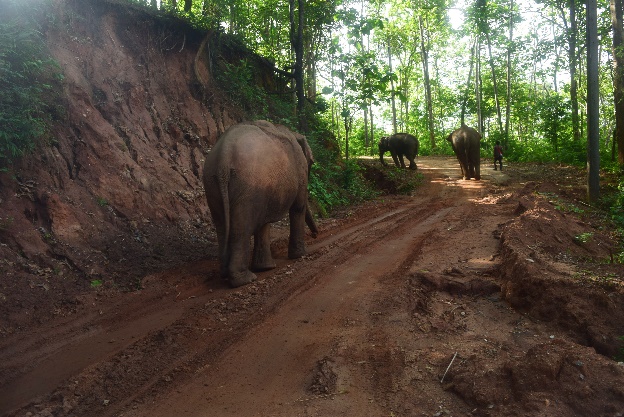


**C.**
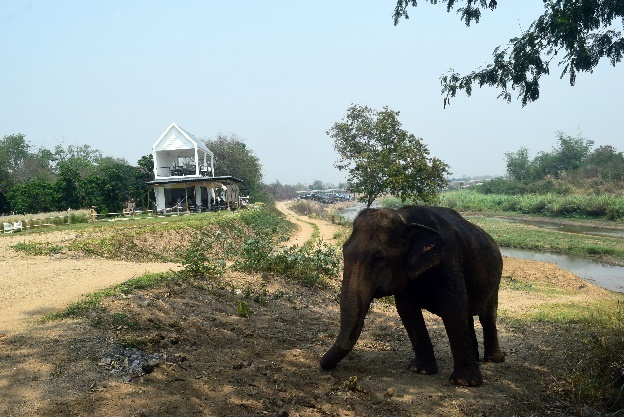
 **D.**
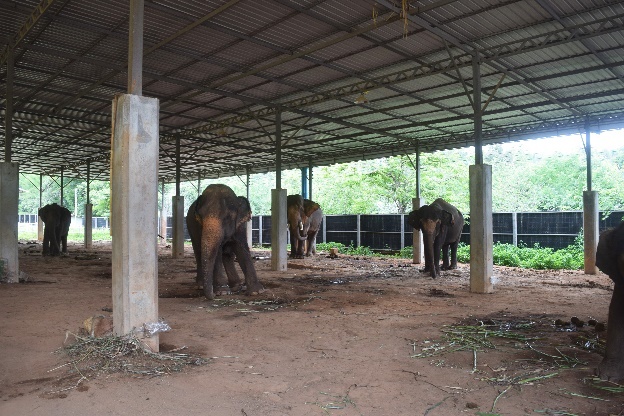


**E.**
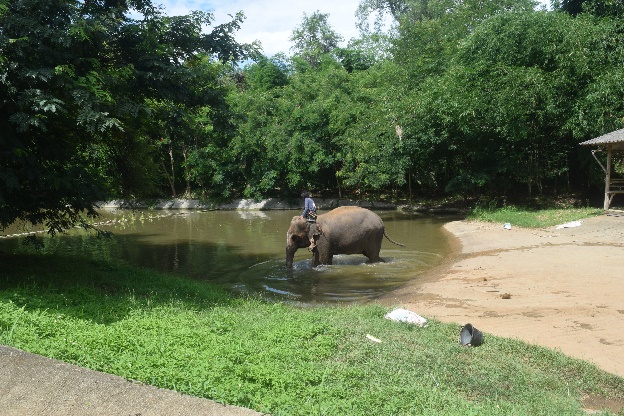
 **F.**
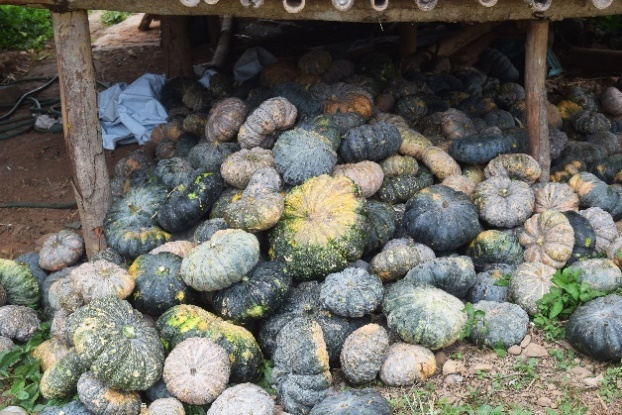


**Supplementary figures 1.** Examples of camp management and mahout routine work during COVID-19. (A) Elephant in a nearby forest, (B) Walking activity, (C) Elephant at coffee café, (D) Covered shed with elephants chained near each other (E) Bathing elephant by mahout (F) Supplement food from private donations. Photography by Jarawee Supanta

**A.**

**B.**

**C.**

**D.**

**Supplementary Figure 2.** Changes in the number of visitors (A), elephants (B), mahouts (C) and other staff (D) at individual elephant tourist camps in northern Thailand before (T00) and through six survey periods [T01 (Apr-Aug 2020), T02 (Sep-Dec, 2020), T3 (Jan-Apr, 2021), T04 (May-Aug 2021), T05 (Sep-Dec, 2021) and T06 (Jan-Apr, 2022)] during the COVID-19 pandemic and corresponding reduction in tourist numbers.

**A.**

**B.**

**Supplementary Figure 3.** Changing trends of food provided (A) roughage (B) supplement at individual elephant tourist camps in northern Thailand before (T00) and through six survey periods [T01 (Apr-Aug 2020), T02 (Sep-Dec, 2020), T3 (Jan-Apr, 2021), T04 (May-Aug 2021), T05 (Sep-Dec, 2021) and T06 (Jan-Apr, 2022)] during the COVID-19 pandemic. Types of high energy treats are described in Figure 5B.

**Supplementary Figure 4.** Mahout activities of the daily routine at individual elephant tourist camps in northern Thailand through six survey periods [T01 (Apr-Aug 2020), T02 (Sep-Dec, 2020), T3 (Jan-Apr, 2021), T04 (May-Aug 2021), T05 (Sep-Dec, 2021) and T06 (Jan-Apr, 2022)] during the COVID-19 pandemic

**3. Supplementary Table 1.** Questionnaire of Project: An assessment of the elephant camp management in the COVID-19 crisis for better health on elephant welfare in Chiang Mai tourist industry. The full questionnaire sheet used to record information during camp visits.
