# Supplementary material for: Effect of the COVID-19 pandemic and international travel ban on elephant tourist camp management in northern Thailand
Source: Front Vet Sci. 2022 Dec 2;9:1038855. doi: 10.3389/fvets.2022.1038855 (PMC9755861; doi:10.3389/fvets.2022.1038855)
Supplement: Supplementary Table 1 — Questionnaire of Project: An assessment of the elephant camp management in the COVID-19 crisis for better health on elephant welfare in Chiang Mai tourist industry. The full questionnaire sheet used to record information during camp visits. [file Table_1.docx]

**แบบสอบถามโครงการการวิเคราะห์การจัดการปางช้างในช่วงสถานการณ์การระบาดของโรคโควิด-19 เพื่อสวัสดิภาพที่ดีด้านสุขภาพของช้างเลี้ยงในธุรกิจการท่องเที่ยวเขตจังหวัดเชียงใหม่**

**Questionnaire of Project: An assessment of the elephant camp management in the COVID-19 crisis for better health on elephant welfare in Chiang Mai tourist industry**

ผู้ให้สัมภาษณ์ (Interviewee)………………………………………………………………………………………………………………………………………………………………………………………………

ผู้สัมภาษณ์ (Interviewer)………………………………………………………………………………………………………………………………………………………………………………………………….

วันที่สัมภาษณ์ (Date)……………………………………………………………………………………………………………………………………………………………………………………………………...

|  | **ก่อนCOVID-19**  **(Before COVID-19)** | **ระหว่างCOVID-19**  **(During COVID-19)** | **หมายเหตุ**  **(Note)** |
| --- | --- | --- | --- |
| 1. ข้อมูลเบื้องต้น (general information) | 1.1 ชื่อปาง (name)…………………………………………………  1.2 ระยะเวลาเปิดกิจการ (How long have the camp been started?).......................  1.3 เขตอำเภอ (district): □ แม่แตง (Mae taeng) □ แม่ริม (Mae rim)  □ แม่วาง (Mae wang) □ หางดง (Hang dong) □ สะเมิง (Samoeng)  □ แม่แจ่ม (Mae Chaem) □ อื่นๆ (others) ………………………..  1.4 กิจกรรมในปางช้าง (camp activity)  □ นั่งหลังช้างแบบใส่แหย่ง (riding saddle)  □ เดินไปกับช้าง (no-riding)  □ นั่งหลังช้างแบบไม่ใส่แหย่ง (bareback)  □ ชมช้าง (observation)  □ อาบน้ำช้างโดยนักท่องเที่ยว (elephant bathing by tourist)  □ การแสดงความสามารถของช้าง (elephant show)  □ ป้อนอาหารช้าง (elephant feeding by tourist)  □ เดินป่า (trekking)  □ อื่นๆ (others) ………………………..  1.5 กิจกรรมที่ไม่เกี่ยวกับช้าง (other activities)  □ ล่องแพ (rafting)  □ ร้านอาหาร (restaurant)  □ ร้านขายของที่ระลึก (gift shop)  □ หมู่บ้านชนเผ่า (tribal village)  1.6 นักท่องเที่ยว (tourists information at least 3 months ago)  □ จำนวนต่อวัน (amount of tourist per day)  …………………………………………………………………………………  …………………………………………………………………………………  …………………………………………………………………………………  …………………………………………………………………………………  □ สัญชาติ (nationality)…………………………………………………………… | 1.1 ชื่อปาง (name)…………………………………………………  1.2 ระยะเวลาเปิดกิจการ (How long have the camp been started?).........................  1.3 วันที่ปิดกิจการช่วงโควิด-19 (When camp closed in COVID-19 period?)  ………………………………………………………………………………  1.3 เขตอำเภอ (district): □ แม่แตง (Mae taeng) □ แม่ริม (Mae rim)  □ แม่วาง (Mae wang) □ หางดง (Hang dong) □ สะเมิง (Samoeng) □ แม่แจ่ม (Mae Chaem) □ อื่นๆ (others) ………………………..  1.4 กิจกรรมในปางช้าง (camp activity)  □ นั่งหลังช้างแบบใส่แหย่ง (riding saddle)  □ เดินไปกับช้าง (no-riding)  □ นั่งหลังช้างแบบไม่ใส่แหย่ง (bareback)  □ ชมช้าง (observation)  □ อาบน้ำช้างโดยนักท่องเที่ยว (elephant bathing by tourist)  □ การแสดงความสามารถของช้าง (elephant show)  □ ป้อนอาหารช้าง (elephant feeding by tourist)  □ เดินป่า (trekking)  □ อื่นๆ (others) ………………………..  1.5 กิจกรรมที่ไม่เกี่ยวกับช้าง (other activities)  □ ล่องแพ (rafting)  □ ร้านอาหาร (restaurant)  □ ร้านขายของที่ระลึก (gift shop)  □ หมู่บ้านชนเผ่า (tribal village)  1.6 นักท่องเที่ยว (tourists information at least 3 months ago)  □ จำนวนต่อวัน (amount of tourist per day)  …………………………………………………………………………………  …………………………………………………………………………………  …………………………………………………………………………………  …………………………………………………………………………………  □ สัญชาติ (nationality)…………………………………………………………… |  |
| 2. ข้อมูลบุคลากร (personal information) | 2.1 จำนวนบุคลากรในปาง (How many people are there in elephant camp?)  …………………………………………………………………………………  2.2 สภาพทางการเงิน (Economic status)   \| ตำแหน่ง (position) / อาชีพหลัก (main job) \| จำนวน (How many?) \| เงินเดือนเฉลี่ยต่อคน (salary per person) \| \| --- \| --- \| --- \| \| เจ้าของปาง (camp owner) \|  \|  \| \| ผู้จัดการ(camp manager) \|  \|  \| \| สัตวแพทย์ประจำปาง (resident veterinarian) \|  \|  \| \| ควาญ (mahout) \|  \|  \| \| พนักงานทำความสะอาด (cleaner) \|  \|  \| \| พนักงานต้อนรับ (officer) \|  \|  \| \| พนักงานขายของที่ระลึก (gift shopkeeper) \|  \|  \| \| พนักงานร้านกาแฟ (coffee shop) \|  \|  \| \| อื่นๆ \|  \|  \|   2.2.1 รายได้เฉลี่ยปาง (Average income):  □ รายวัน (Daily)........................................บาท/วัน (baht per day)  □ รายเดือน (Monthly)...............................บาท/เดือน (baht per month)  2.2.2 อาชีพเสริมของปาง (Part-time):  □ ไม่มี (No)  □ มี (Yes): ระบุ (What?)......................................................................  รายได้ของอาชีพเสริม (Income of part-time):  □ รายวัน (Daily)........................................บาท/วัน (baht per day)  □ รายเดือน (Monthly)...............................บาท/เดือน (baht per month)  2.2.3 รายได้รวมเฉลี่ยต่อเดือน ของอาชีพเสริม  (Total income per month of part-time).......................................บาท (baht) | 2.1 จำนวนบุคลากรในปาง (How many people are there in elephant camp?)  …………………………………………………………………………………..  2.2 สภาพทางการเงิน (Economic status)   \| ตำแหน่ง (position) / อาชีพหลัก (main job) \| จำนวน (How many?) \| เงินเดือนเฉลี่ยต่อคน (salary per person) \| \| --- \| --- \| --- \| \| เจ้าของปาง (camp owner) \|  \|  \| \| ผู้จัดการ(camp manager) \|  \|  \| \| สัตวแพทย์ประจำปาง (resident veterinarian) \|  \|  \| \| ควาญ (mahout) \|  \|  \| \| พนักงานทำความสะอาด (cleaner) \|  \|  \| \| พนักงานต้อนรับ (officer) \|  \|  \| \| พนักงานขายของที่ระลึก (gift shopkeeper) \|  \|  \| \| พนักงานร้านกาแฟ (coffee shop) \|  \|  \| \| อื่นๆ \|  \|  \|  \| สาเหตุ (cause) \| เพิ่มขึ้น (increase) \| ลดลง (decrease) \| \| --- \| --- \| --- \| \| จำนวนพนักงาน  (no. of person) \|  \|  \| \| เงินเดือน (salary) \|  \|  \|   2.2.1 รายได้เฉลี่ยปาง (Average income):  □ รายวัน (Daily)........................................บาท/วัน (baht per day)  □ รายเดือน (Monthly)...............................บาท/เดือน (baht per month)  2.2.2 อาชีพเสริมของปาง (Part-time ):  □ ไม่มี (No)  □ มี (Yes): ระบุ (What?)......................................................................  รายได้ของอาชีพเสริม (Income of part-time):  □ รายวัน (Daily)........................................บาท/วัน (baht per day)  □ รายเดือน (Monthly)...............................บาท/เดือน (baht per month)  2.2.3 รายได้รวมเฉลี่ยต่อเดือน ของอาชีพเสริม  (Total income per month of part-time).......................................บาท (baht) |  |
| 3. ข้อมูลช้าง (elephant information) | 3.1 จำนวนช้าง (elephant number)………………………เชือก (elephants)   \| เพศผู้ (male) \| ระบุชื่อ/อายุ \| จำนวน (number) \| \| --- \| --- \| --- \| \| □ 0 -3 ปี (yrs) \|  \|  \| \| □ 4-10 ปี (yrs) \|  \|  \| \| □ 11-20 ปี (yrs) \|  \|  \| \| □ 21-30 ปี (yrs) \|  \|  \| \| □ 31-40 ปี (yrs) \|  \|  \| \| □ 41-55 ปี (yrs) \|  \|  \| \| □ >55 ปี (yrs) \|  \|  \|  \| เพศเมีย (female) \| ระบุชื่อ/อายุ \| จำนวน (number) \| \| --- \| --- \| --- \| \| □ 0 -3 ปี (yrs) \|  \|  \| \| □ 4-10 ปี (yrs) \|  \|  \| \| □ 11-20 ปี (yrs) \|  \|  \| \| □ 21-30 ปี (yrs) \|  \|  \| \| □ 31-40 ปี (yrs) \|  \|  \| \| □ 41-55 ปี (yrs) \|  \|  \| \| □ >55 ปี (yrs) \|  \|  \|   3.2 แหล่งที่มาของช้าง (source of elephant)  ............................................................................................................................  3.3 การครอบครองช้าง (owner of elephant)  □ เป็นของเจ้าของปางช้าง (camp own)………………………เชือก (elephants)  □ เป็นช้างเช่า (rent):  □ เป็นของควาญเอง (mahout own)………………………เชือก (elephants)  □ เป็นของบุคคลอื่น (other person own)…………………เชือก (elephants)  3.4 อัตราส่วนช้างต่อควาญ (elephant: mahout ratio)  ............................................................................................................................ | 3.1 จำนวนช้าง (elephant number)………………………เชือก (elephants)   \| เพศผู้ (male) \| ระบุชื่อ/อายุ \| จำนวน (number) \| \| --- \| --- \| --- \| \| □ 0 -3 ปี (yrs) \|  \|  \| \| □ 4-10 ปี (yrs) \|  \|  \| \| □ 11-20 ปี (yrs) \|  \|  \| \| □ 21-30 ปี (yrs) \|  \|  \| \| □ 31-40 ปี (yrs) \|  \|  \| \| □ 41-55 ปี (yrs) \|  \|  \| \| □ >55 ปี (yrs) \|  \|  \|  \| เพศเมีย (female) \| ระบุชื่อ/อายุ \| จำนวน (number) \| \| --- \| --- \| --- \| \| □ 0 -3 ปี (yrs) \|  \|  \| \| □ 4-10 ปี (yrs) \|  \|  \| \| □ 11-20 ปี (yrs) \|  \|  \| \| □ 21-30 ปี (yrs) \|  \|  \| \| □ 31-40 ปี (yrs) \|  \|  \| \| □ 41-55 ปี (yrs) \|  \|  \| \| □ >55 ปี (yrs) \|  \|  \|   3.2 แหล่งที่มาของช้าง (source of elephant)  ..............................................................................................................................  3.3 การครอบครองช้าง (owner of elephant)  □ เป็นของเจ้าของปางช้าง (camp own)………………………เชือก (elephants)  □ เป็นช้างเช่า (rent):  □ เป็นของควาญเอง (mahout own)………………………เชือก (elephants)  □ เป็นของบุคคลอื่น (other person own)…………………เชือก (elephants)  3.4 อัตราส่วนช้างต่อควาญ (elephant: mahout ratio)  ..............................................................................................................................  3.3 สาเหตุที่จำนวนช้างที่เปลี่ยนแปลง  □ เพิ่มขึ้น (increase) เพราะ (because)…………………………………………..  รับมาจากที่ไหน (where)……………………………………………………...  □ ลดลง (decrease) เพราะ (because)....................................................................  □ ย้าย (move)………………..กี่เชือก (elephant)  ย้ายไปที่ไหน (where)…………………………………………………….  ลักษณะการเลี้ยงดูในสถานที่ใหม่ (what is the characteristic of new camp or location?)……………………………………………………………………...  …………………………………………………………………………………...  □ ขาย (sold) ตัวละเท่าไหร่ (how much?)  □ ปิดปาง (closed)  สาเหตุที่ปิดปาง (cause)   - 1. มีแนวโน้มที่จะกลับมาเปิดปางอีกไหม จะเปิดเมื่อไหร่ (when camp will operate?)   ……………………………………………………………………………….. |  |
| 4. การจัดการด้านการทำงานของช้าง (working management) | 4.1 ตารางการทำงานช้าง (Elephant work schedule)  ……………………………………………………………………………………….  ……………………………………………………………………………………….  ……………………………………………………………………………………….  ……………………………………………………………………………………….  ……………………………………………………………………………………….  ……………………………………………………………………………………….  ……………………………………………………………………………………….  ……………………………………………………………………………………….  ……………………………………………………………………………………….  ……………………………………………………………………………………….  ……………………………………………………………………………………….  ……………………………………………………………………………………….  4.2 ช่วงเวลาที่ช้างทำงาน (period of working)  เวลาเริ่มงาน (time to start).................................................  เวลาเลิกงาน (time to stop).................................................  4.3 ช่วงเวลาพักระหว่างวัน (break time)  □ไม่มี (no)  □ มี (yes) โปรดระบุ (please specify)......................................................................  4.4 อุปกรณ์ที่ใช้จับบังคับช้างขณะทำงาน (restraint equipment)  □ไม่มี (no)  □ มี (yes):  ประเภทของอุปกรณ์จับบังคับ (type of restraint equipment)  □ ตะขอ (hook)  □ ตะปู (nail)  □ มีด (knife)  □ หนังสติ๊ก (slingshot)  □ โซ่ (chain)  □ อื่นๆ (others).....................  ควาญใช้อุปกรณ์จับบังคับเมื่อใด (when mahout use the restraint equipment?)  …………………………………………………………………………………  4.2 ช้างที่มีการเดินตามเส้นทาง (trekking elephant)  4.2.1 ช้างเดินวันละกี่รอบ (How many rounds per day?) ............รอบ (rounds)  4.2.2 ช้างเดินเป็นระยะทางเท่าไหร่ต่อรอบ  (How many kilometers of the track per round?) ...........กิโลเมตร (kilometers)  4.2.3 ช้างเดินนานเท่าไหร่ต่อรอบ  (How long does it take for 1 round?) ..........................นาที (minutes)  4.2.4 ช้างเดินด้วยอัตราเท่าไหร่ (how much walking rate?)  …………………………………………..…..กิโลเมตรต่อชั่วโมง (km/hr)  4.3 ปัญหาที่เกิดจากการทำกิจกรรม (problem after work)  □ไม่มี (no)  □ มี (yes):  สาเหตุของการได้รับบาดเจ็บ (cause of injury)  □ อุปกรณ์ควบคุม (restraint equipment)  โปรดระบุ (please specify)........................................................................  □ อุปกรณ์ใส่แหย่ง (saddle equipment)  □ เส้นทางการเดินไม่เหมาะสม (improper pathway)  □ ช้างทำร้ายกัน (elephant attack)  □ อ่อนเพลียจากการทำงานหนัก (weakness from hard work)  □ อื่นๆ (others)…………………………………………………..……… | 4.1 ตารางการทำงานช้าง (Elephant work schedule)  ……………………………………………………………………………………  ……………………………………………………………………………………  ……………………………………………………………………………………  ……………………………………………………………………………………  ……………………………………………………………………………………  ……………………………………………………………………………………  ………………………………………………………………………………………………………………………………………………………………………………………………………………………………………………………………………………………………………………………………………………………………………………………………………………………………………………………………………………………………………………………………  4.2 ช่วงเวลาที่ช้างทำงาน (period of working)  เวลาเริ่มงาน (time to start).................................................  เวลาเลิกงาน (time to stop).................................................  4.3 ช่วงเวลาพักระหว่างวัน (break time)  □ไม่มี (no)  □ มี (yes) โปรดระบุ (please specify)......................................................................  4.4 อุปกรณ์ที่ใช้จับบังคับช้างขณะทำงาน (restraint equipment)  □ไม่มี (no)  □ มี (yes):  ประเภทของอุปกรณ์จับบังคับ (type of restraint equipment)  □ ตะขอ (hook)  □ ตะปู (nail)  □ มีด (knife)  □ หนังสติ๊ก (slingshot)  □ โซ่ (chain)  □ อื่นๆ (others).....................  ควาญใช้อุปกรณ์จับบังคับเมื่อใด (when mahout use the restraint equipment?)  …………………………………………………………………………………...  4.2 ช้างที่มีการเดินตามเส้นทาง (trekking elephant)  4.2.1 ช้างเดินวันละกี่รอบ (How many rounds per day?) ...............รอบ (rounds)  4.2.2 ช้างเดินเป็นระยะทางเท่าไหร่ต่อรอบ  (How many kilometers of the track per round?) .............กิโลเมตร (kilometers)  4.2.3 ช้างเดินนานเท่าไหร่ต่อรอบ  (How long does it take for 1 round?) ..........................นาที (minutes)  4.2.4 ช้างเดินด้วยอัตราเท่าไหร่ (how much walking rate?)  ……………………………………………………..กิโลเมตรต่อชั่วโมง (km/hr)  4.3 ปัญหาที่เกิดจากการทำกิจกรรม (problem after work)  □ไม่มี (no)  □ มี (yes):  สาเหตุของการได้รับบาดเจ็บ (cause of injury)  □ อุปกรณ์ควบคุม (restraint equipment)  โปรดระบุ (please specify)........................................................................  □ อุปกรณ์ใส่แหย่ง (saddle equipment)  □ เส้นทางการเดินไม่เหมาะสม (improper pathway)  □ ช้างทำร้ายกัน (elephant attack)  □ อ่อนเพลียจากการทำงานหนัก (weakness from hard work)  □ อื่นๆ (others)………………………………………………………………… |  |
| 5.การจัดการทางโภชนาการ (nutrition management) | 5.1 อาหารที่ให้ช้างเป็นประจำ (elephant food)  5.1.1 อาหารหยาบ (roughage)  □ หญ้าเนเปียร์ (Napier grass) □ ต้นสัปปะรด (pineapple stalk )  □ กล้วย (banana) □ หญ้าบาน่า (Bana grass)  □ ต้นไผ่ (bamboo) □ อ้อย (sugar cane)  □ ต้นข้าวโพด (corn stalk) □ หญ้าแห้ง (hey)  □ ผักหรือผลไม้ (vegetable/fruit) โปรดระบุ (please specify)….  □ อาหารในป่า (food in forest)  □ หญ้าพื้นถิ่น (local roughage) โปรดระบุ (please specify)............................   \| อาหารหยาบ (roughage) \| แหล่งที่มา (source) \| ความสด (freshness) \| การเก็บรักษา(storage) \| \| --- \| --- \| --- \| --- \| \|  \|  \|  \|  \|   5.1.2 อาหารข้น (concentrate food)  □ ไม่ให้ (no)  □ ให้ (yes): □ อาหารเม็ดสำหรับม้า (pellet food for horse)  □ อาหารเม็ดสำหรับช้าง (pellet food elephant)  □ อื่นๆ (others).................................................................................................   \| อาหารข้น (concentrate food) \| แหล่งที่มา (source) \| เปอร์เซนต์โปรตีน (% protein) \| การเก็บรักษา(storage) \| \| --- \| --- \| --- \| --- \| \|  \|  \|  \|  \|   5.1.3 อาหารเสริม (supplement)  □ ไม่ให้ (no)  □ ให้ (yes): □ วิตามินแบบเม็ด (tablet vitamin) □ สมุนไพร (herb)  □ ข้าวเหนียว (sticky rice) □ มะขามเปียก (tamarind)  □ อื่นๆ (others)..................................................................................................   \| อาหารเสริม (supplement) \| แหล่งที่มา (source) \| เมื่อใด/กรณีใด (When) \| การเก็บรักษา(storage) \| \| --- \| --- \| --- \| --- \| \|  \|  \|  \|  \|   5.2 การให้อาหารช้าง  5.2.1 ช้างก่อนหย่านม (prewean)   \|  \| ชนิด (type) \| ปริมาณต่อวัน (amount/day) \| ความถี่ต่อวัน (No. of feeding/ day) \| ปริมาณต่อครั้ง (amount/  feeding) \| \| --- \| --- \| --- \| --- \| --- \| \| อาหารหยาบ(roughage) \|  \|  \|  \|  \| \| อาหารข้น(concentrate) \|  \|  \|  \|  \| \| อาหารเสริม (supplement) \|  \|  \|  \|  \|   5.2.2 ช้างหลังหย่านม - 10 ปี (wean - 10 yrs)   \|  \| ชนิด (type) \| ปริมาณต่อวัน (amount/day) \| ความถี่ต่อวัน (No. of feeding/ day) \| ปริมาณต่อครั้ง (amount/  feeding) \| \| --- \| --- \| --- \| --- \| --- \| \| อาหารหยาบ(roughage) \|  \|  \|  \|  \| \| อาหารข้น(concentrate) \|  \|  \|  \|  \| \| อาหารเสริม (supplement) \|  \|  \|  \|  \|   5.2.3 ช้างโตเพศผู้ (male adult)   \|  \| ชนิด (type) \| ปริมาณต่อวัน (amount/day) \| ความถี่ต่อวัน (No. of feeding/ day) \| ปริมาณต่อครั้ง (amount/  feeding) \| \| --- \| --- \| --- \| --- \| --- \| \| อาหารหยาบ(roughage) \|  \|  \|  \|  \| \| อาหารข้น(concentrate) \|  \|  \|  \|  \| \| อาหารเสริม (supplement) \|  \|  \|  \|  \|   5.2.4 ช้างโตเพศเมีย (female adult)   \|  \| ชนิด (type) \| ปริมาณต่อวัน (amount/day) \| ความถี่ต่อวัน (No. of feeding/ day) \| ปริมาณต่อครั้ง (amount/  feeding) \| \| --- \| --- \| --- \| --- \| --- \| \| อาหารหยาบ(roughage) \|  \|  \|  \|  \| \| อาหารข้น(concentrate) \|  \|  \|  \|  \| \| อาหารเสริม (supplement) \|  \|  \|  \|  \|   5.2.5 ช้างชรา 55 ปีขึ้นไป (old elephant: > 55 yrs)   \|  \| ชนิด (type) \| ปริมาณต่อวัน (amount/day) \| ความถี่ต่อวัน (No. of feeding/ day) \| ปริมาณต่อครั้ง (amount/  feeding) \| \| --- \| --- \| --- \| --- \| --- \| \| อาหารหยาบ(roughage) \|  \|  \|  \|  \| \| อาหารข้น(concentrate) \|  \|  \|  \|  \| \| อาหารเสริม (supplement) \|  \|  \|  \|  \|   5.2.6 ช้างตกมัน (musth elephant)   \|  \| ชนิด (type) \| ปริมาณต่อวัน (amount/day) \| ความถี่ต่อวัน (No. of feeding/ day) \| ปริมาณต่อครั้ง (amount/  feeding) \| \| --- \| --- \| --- \| --- \| --- \| \| อาหารหยาบ(roughage) \|  \|  \|  \|  \| \| อาหารข้น(concentrate) \|  \|  \|  \|  \| \| อาหารเสริม (supplement) \|  \|  \|  \|  \|   5.2.7 ช้างท้อง (pregnant elephant)  - ระยะแรก (early)   \|  \| ชนิด (type) \| ปริมาณต่อวัน (amount/day) \| ความถี่ต่อวัน (No. of feeding/ day) \| ปริมาณต่อครั้ง (amount/  feeding) \| \| --- \| --- \| --- \| --- \| --- \| \| อาหารหยาบ(roughage) \|  \|  \|  \|  \| \| อาหารข้น(concentrate) \|  \|  \|  \|  \| \| อาหารเสริม (supplement) \|  \|  \|  \|  \|   - ระยะกลาง (mid)   \|  \| ชนิด (type) \| ปริมาณต่อวัน (amount/day) \| ความถี่ต่อวัน (No. of feeding/ day) \| ปริมาณต่อครั้ง (amount/  feeding) \| \| --- \| --- \| --- \| --- \| --- \| \| อาหารหยาบ(roughage) \|  \|  \|  \|  \| \| อาหารข้น(concentrate) \|  \|  \|  \|  \| \| อาหารเสริม (supplement) \|  \|  \|  \|  \|   - ระยะท้าย (late)   \|  \| ชนิด (type) \| ปริมาณต่อวัน (amount/day) \| ความถี่ต่อวัน (No. of feeding/ day) \| ปริมาณต่อครั้ง (amount/  feeding) \| \| --- \| --- \| --- \| --- \| --- \| \| อาหารหยาบ(roughage) \|  \|  \|  \|  \| \| อาหารข้น(concentrate) \|  \|  \|  \|  \| \| อาหารเสริม (supplement) \|  \|  \|  \|  \|   อื่นๆ (others)  ……………………………………………………………………………………..……………………………………………………………………………………..…………………………………………………………………………………….. | 5.1 อาหารที่ให้ช้างเป็นประจำ (elephant food)  5.1.1 อาหารหยาบ (roughage)  □ หญ้าเนเปียร์ (Napier grass) □ ต้นสัปปะรด (pineapple stalk )  □ กล้วย (banana) □ หญ้าบาน่า (Bana grass)  □ ต้นไผ่ (bamboo) □ อ้อย (sugar cane)  □ ต้นข้าวโพด (corn stalk) □ หญ้าแห้ง (hey)  □ ผักหรือผลไม้ (vegetable/fruit) โปรดระบุ (please specify)….  □ อาหารในป่า (food in forest)  □ หญ้าพื้นถิ่น (local roughage) โปรดระบุ (please specify)................................   \| อาหารหยาบ (roughage) \| แหล่งที่มา (source) \| ความสด (freshness) \| การเก็บรักษา(storage) \| \| --- \| --- \| --- \| --- \| \|  \|  \|  \|  \|   5.1.2 อาหารข้น (concentrate food)  □ ไม่ให้ (no)  □ ให้ (yes): □ อาหารเม็ดสำหรับม้า (pellet food for horse)  □ อาหารเม็ดสำหรับช้าง (pellet food elephant)  □ อื่นๆ (others)....................................................................................................   \| อาหารข้น (concentrate food) \| แหล่งที่มา (source) \| เปอร์เซนต์โปรตีน (% protein) \| การเก็บรักษา(storage) \| \| --- \| --- \| --- \| --- \| \|  \|  \|  \|  \|   5.1.3 อาหารเสริม (supplement)  □ ไม่ให้ (no)  □ ให้ (yes): □ วิตามินแบบเม็ด (tablet vitamin) □ สมุนไพร (herb)  □ ข้าวเหนียว (sticky rice) □ มะขามเปียก (tamarind)  □ อื่นๆ (others)....................................................................................................   \| อาหารเสริม (supplement) \| แหล่งที่มา (source) \| เมื่อใด/กรณีใด (When) \| การเก็บรักษา(storage) \| \| --- \| --- \| --- \| --- \| \|  \|  \|  \|  \|   5.2 การให้อาหารช้าง  5.2.1 ช้างก่อนหย่านม (prewean)   \|  \| ชนิด (type) \| ปริมาณต่อวัน (amount/day) \| ความถี่ต่อวัน (No. of feeding/ day) \| ปริมาณต่อครั้ง (amount/  feeding) \| \| --- \| --- \| --- \| --- \| --- \| \| อาหารหยาบ(roughage) \|  \|  \|  \|  \| \| อาหารข้น(concentrate) \|  \|  \|  \|  \| \| อาหารเสริม (supplement) \|  \|  \|  \|  \|   5.2.2 ช้างหลังหย่านม - 10 ปี (wean - 10 yrs)   \|  \| ชนิด (type) \| ปริมาณต่อวัน (amount/day) \| ความถี่ต่อวัน (No. of feeding/ day) \| ปริมาณต่อครั้ง (amount/  feeding) \| \| --- \| --- \| --- \| --- \| --- \| \| อาหารหยาบ(roughage) \|  \|  \|  \|  \| \| อาหารข้น(concentrate) \|  \|  \|  \|  \| \| อาหารเสริม (supplement) \|  \|  \|  \|  \|   5.2.3 ช้างโตเพศผู้ (male adult)   \|  \| ชนิด (type) \| ปริมาณต่อวัน (amount/day) \| ความถี่ต่อวัน (No. of feeding/ day) \| ปริมาณต่อครั้ง (amount/  feeding) \| \| --- \| --- \| --- \| --- \| --- \| \| อาหารหยาบ(roughage) \|  \|  \|  \|  \| \| อาหารข้น(concentrate) \|  \|  \|  \|  \| \| อาหารเสริม (supplement) \|  \|  \|  \|  \|   5.2.4 ช้างโตเพศเมีย (female adult)   \|  \| ชนิด (type) \| ปริมาณต่อวัน (amount/day) \| ความถี่ต่อวัน (No. of feeding/ day) \| ปริมาณต่อครั้ง (amount/  feeding) \| \| --- \| --- \| --- \| --- \| --- \| \| อาหารหยาบ(roughage) \|  \|  \|  \|  \| \| อาหารข้น(concentrate) \|  \|  \|  \|  \| \| อาหารเสริม (supplement) \|  \|  \|  \|  \|   5.2.5 ช้างชรา 55 ปีขึ้นไป (old elephant: > 55 yrs)   \|  \| ชนิด (type) \| ปริมาณต่อวัน (amount/day) \| ความถี่ต่อวัน (No. of feeding/ day) \| ปริมาณต่อครั้ง (amount/  feeding) \| \| --- \| --- \| --- \| --- \| --- \| \| อาหารหยาบ(roughage) \|  \|  \|  \|  \| \| อาหารข้น(concentrate) \|  \|  \|  \|  \| \| อาหารเสริม (supplement) \|  \|  \|  \|  \|   5.2.6 ช้างตกมัน (musth elephant)   \|  \| ชนิด (type) \| ปริมาณต่อวัน (amount/day) \| ความถี่ต่อวัน (No. of feeding/ day) \| ปริมาณต่อครั้ง (amount/  feeding) \| \| --- \| --- \| --- \| --- \| --- \| \| อาหารหยาบ(roughage) \|  \|  \|  \|  \| \| อาหารข้น(concentrate) \|  \|  \|  \|  \| \| อาหารเสริม (supplement) \|  \|  \|  \|  \|   5.2.7 ช้างท้อง (pregnant elephant)  - ระยะแรก (early)   \|  \| ชนิด (type) \| ปริมาณต่อวัน (amount/day) \| ความถี่ต่อวัน (No. of feeding/ day) \| ปริมาณต่อครั้ง (amount/  feeding) \| \| --- \| --- \| --- \| --- \| --- \| \| อาหารหยาบ(roughage) \|  \|  \|  \|  \| \| อาหารข้น(concentrate) \|  \|  \|  \|  \| \| อาหารเสริม (supplement) \|  \|  \|  \|  \|   - ระยะกลาง (mid)   \|  \| ชนิด (type) \| ปริมาณต่อวัน (amount/day) \| ความถี่ต่อวัน (No. of feeding/ day) \| ปริมาณต่อครั้ง (amount/  feeding) \| \| --- \| --- \| --- \| --- \| --- \| \| อาหารหยาบ(roughage) \|  \|  \|  \|  \| \| อาหารข้น(concentrate) \|  \|  \|  \|  \| \| อาหารเสริม (supplement) \|  \|  \|  \|  \|   - ระยะท้าย (late)   \|  \| ชนิด (type) \| ปริมาณต่อวัน (amount/day) \| ความถี่ต่อวัน (No. of feeding/ day) \| ปริมาณต่อครั้ง (amount/  feeding) \| \| --- \| --- \| --- \| --- \| --- \| \| อาหารหยาบ(roughage) \|  \|  \|  \|  \| \| อาหารข้น(concentrate) \|  \|  \|  \|  \| \| อาหารเสริม (supplement) \|  \|  \|  \|  \|   อื่นๆ (others)  ……………………………………………………………………………………..……………………………………………………………………………………..…………………………………………………………………………………….. |  |
| 6. น้ำบริโภค (drinking water) | \|  \| กลางวัน(Day time) \| กลางคืน(Night time) \| \| --- \| --- \| --- \| \| แหล่งที่มา (source) \| □ แม่น้ำ (river)  □ บ่อน้ำ (pond)  □ น้ำบาดาล (underground water)  □ น้ำประปา (tap water) \| □ แม่น้ำ (river)  □ บ่อน้ำ (pond)  □ น้ำบาดาล (underground water)  □ น้ำประปา (tap water) \| \| ลักษณะจุดให้น้ำบริโภคของช้าง (characteristic of elephant drinking point) \| □ บ่อน้ำที่ก่อขึ้นมา (human-build pond)  □ ก๊อกน้ำ (tap)  □ ถังน้ำ (bucket)  □ แหล่งน้ำธรรมชาติ (natural water)  □ อื่นๆ (others)............ \| □ บ่อน้ำที่ก่อขึ้นมา (human-build pond)  □ ก๊อกน้ำ (tap)  □ ถังน้ำ (bucket)  □ แหล่งน้ำธรรมชาติ (natural water)  □ อื่นๆ (others)............ \| \| จำนวนจุดที่ช้างสามารถดื่มน้ำได้ (How many points that elephant can access to drink?) \| ...................จุด (points) \| ...................จุด (points) \| \| ช้างได้ดื่มน้ำกี่ครั้งต่อวัน (How many time that elephant can drink water?) \| ......ครั้ง/วัน (time/day)  □ ไม่ได้กำหนด (not fix) \| ......ครั้ง/วัน (time/day)  □ ไม่ได้กำหนด (not fix) \| \| ช้างสามารถดื่มน้ำได้โดยวิธีใดบ้าง (How can elephant drink water?) \| □ ด้วยตนเอง (by itself)  □ ควาญให้/พาไป (by mahout) \| □ ด้วยตนเอง (by itself)  □ ควาญให้/พาไป (by mahout) \| | \|  \| กลางวัน(Day time) \| กลางคืน(Night time) \| \| --- \| --- \| --- \| \| แหล่งที่มา (source) \| □ แม่น้ำ (river)  □ บ่อน้ำ (pond)  □ น้ำบาดาล (underground water)  □ น้ำประปา (tap water) \| □ แม่น้ำ (river)  □ บ่อน้ำ (pond)  □ น้ำบาดาล (underground water)  □ น้ำประปา (tap water) \| \| ลักษณะจุดให้น้ำบริโภคของช้าง (characteristic of elephant drinking point) \| □ บ่อน้ำที่ก่อขึ้นมา (human-build pond)  □ ก๊อกน้ำ (tap)  □ ถังน้ำ (bucket)  □ แหล่งน้ำธรรมชาติ (natural water)  □ อื่นๆ (others)............ \| □ บ่อน้ำที่ก่อขึ้นมา (human-build pond)  □ ก๊อกน้ำ (tap)  □ ถังน้ำ (bucket)  □ แหล่งน้ำธรรมชาติ (natural water)  □ อื่นๆ (others)............ \| \| จำนวนจุดที่ช้างสามารถดื่มน้ำได้ (How many points that elephant can access to drink?) \| ...................จุด (points) \| ...................จุด (points) \| \| ช้างได้ดื่มน้ำกี่ครั้งต่อวัน (How many time that elephant can drink water?) \| ......ครั้ง/วัน (time/day)  □ ไม่ได้กำหนด (not fix) \| ......ครั้ง/วัน (time/day)  □ ไม่ได้กำหนด (not fix) \| \| ช้างสามารถดื่มน้ำได้โดยวิธีใดบ้าง (How can elephant drink water?) \| □ ด้วยตนเอง (by itself)  □ ควาญให้/พาไป (by mahout) \| □ ด้วยตนเอง (by itself)  □ ควาญให้/พาไป (by mahout) \| |  |
| 7. การจัดการที่พักสำหรับช้าง (rest area management) | 7.1 ประเภทของที่พักช้าง (type of rest area)  □ ป่าธรรมชาติ (forest): □ มัด (chain) □ ปล่อย (free)  □ สวน (field) : □ มัด (chain)  □ ปล่อย (free)  □ ให้อยู่ในที่โล่งภายในปาง (outdoor in camp) : □ มัด (chain)  □ ปล่อย (free)  □ ล่ามไว้ใต้ต้นไม้ (chain under tree)  □ ล่ามไว้โรงเรือน (chain in building) : □ มัด (chain)  □ ปล่อย (free)  □ คอก (corral) : □ มัด (chain)  □ ปล่อย (free)  7.2 จำนวนตัวช้างต่อประเภทที่พักช้าง (number of elephant each type of rest area)  ..........................................................................ตัวต่อที่พัก (elephant per rest area)  7.3 การล่ามช้างในที่พักช้าง (elephant chain in rest area)  7.3.1 มีการล่ามช้างในที่พักช้างหรือไม่ (Do they chain elephant in rest area?)  □ไม่มี (no)  □ มี (yes)  7.3.2 มีการล่ามช้างทุกเชือกในที่พักช้างหรือไม่ (Are all elephant chained in rest area?) □ ทุกเชือก (all)  □ บางเชือก (some)  7.3.3 มีการล่ามช้างในที่พักช้างช่วงใดบ้าง (When do they chain elephant in rest area?) □ ตลอดเวลา (all the time)  □ ช่วงที่ไม่ได้ทำงาน (during no work)  □ ช่วงกลางคืน (nighttime)  7.3.4 โซ่ (chain)  ตัวผู้ (male)  ความยาวโซ่ (length of chain)……………………เมตร (meters)  ขนาดโซ่ (size of chain) ....................................................หุน (units)  ตัวเมีย (female)  ความยาวโซ่ (length of chain)……………………เมตร (meters)  ขนาดโซ่ (size of chain) ....................................................หุน (units) | 7.1 ประเภทของที่พักช้าง (type of rest area)  □ ป่าธรรมชาติ (forest): □ มัด (chain) □ ปล่อย (free)  □ สวน (field) : □ มัด (chain)  □ ปล่อย (free)  □ ให้อยู่ในที่โล่งภายในปาง (outdoor in camp) : □ มัด (chain)  □ ปล่อย (free)  □ ล่ามไว้ใต้ต้นไม้ (chain under tree)  □ ล่ามไว้โรงเรือน (chain in building) : □ มัด (chain)  □ ปล่อย (free)  □ คอก (corral) : □ มัด (chain)  □ ปล่อย (free)  7.2 จำนวนตัวช้างต่อประเภทที่พักช้าง (number of elephant each type of rest area)  ..........................................................................ตัวต่อที่พัก (elephant per rest area)  7.3 การล่ามช้างในที่พักช้าง (elephant chain in rest area)  7.3.1 มีการล่ามช้างในที่พักช้างหรือไม่ (Do they chain elephant in rest area?)  □ไม่มี (no)  □ มี (yes)  7.3.2 มีการล่ามช้างทุกเชือกในที่พักช้างหรือไม่ (Are all elephant chained in rest area?) □ ทุกเชือก (all)  □ บางเชือก (some)  7.3.3 มีการล่ามช้างในที่พักช้างช่วงใดบ้าง (When do they chain elephant in rest area?) □ ตลอดเวลา (all the time)  □ ช่วงที่ไม่ได้ทำงาน (during no work)  □ ช่วงกลางคืน (nighttime)  7.3.4 โซ่ (chain)  ตัวผู้ (male)  ความยาวโซ่ (length of chain)……………………เมตร (meters)  ขนาดโซ่ (size of chain) ....................................................หุน (units)  ตัวเมีย (female)  ความยาวโซ่ (length of chain)……………………เมตร (meters)  ขนาดโซ่ (size of chain) ....................................................หุน (units) |  |
| 8. การดูแลสุขภาพ (elephant health management) | 8.1 มีคนดูแลสุขภาพช้างหรือไม่ (resident veterinarian)  □ ไม่มี (no)  □ มี (yes) โปรดระบุ (please specify)………………………………  8.2 การจัดการช้างป่วย (How to manage sick elephant?)  □ ส่งโรงพยาบาลช้างใกล้เคียง (sent to elephant hospital)  □ รักษาเอง (self treatment)  □ อื่นๆ (others)  8.3 โปรแกรมถ่ายพยาธิ ทำวัคซีน (deworm and vaccination)  □ไม่มี (no)  □ มี (yes): ถ่ายพยาธิกี่ครั้งต่อปี (How many times per year?)..............ครั้ง (times)  ถ่ายพยาธิทุกเชือกหรือไม่ (all elephant):  □ ไม่ใช่ (no)  □ ใช่ (yes)  ชนิดของยา (please specify deworming drug)........................................................  8.4 ความถี่การอาบน้ำช้างต่อวัน (frequency of showing per day)  จำนวนครั้ง (times)………………………(times)  ช่วงเวลา (when)…………………………...  ช้างอาบเองหรือไม่ (shower by itself, or not?)  □ ไม่ใช่ (no)  □ ใช่ (yes)  8.5 ปัญหาสุขภาพที่พบ (health problems)  □ไม่มี (no)  □ มี (yes) โปรดระบุ (please specify)………………………………………….  8.6 ลักษณะพฤติกรรมที่เปลี่ยนไป (abnormal behavior)  ………………………………………………………………………………………………………………………………………………………………………… | 8.1 มีคนดูแลสุขภาพช้างหรือไม่ (resident veterinarian)  □ ไม่มี (no)  □ มี (yes) โปรดระบุ (please specify)………………………………  8.2 การจัดการช้างป่วย (How to manage sick elephant?)  □ ส่งโรงพยาบาลช้างใกล้เคียง (sent to elephant hospital)  □ รักษาเอง (self treatment)  □ อื่นๆ (others)  8.3 โปรแกรมถ่ายพยาธิ ทำวัคซีน (deworm and vaccination)  □ไม่มี (no)  □ มี (yes): ถ่ายพยาธิกี่ครั้งต่อปี (How many times per year?)..............ครั้ง (times)  ถ่ายพยาธิทุกเชือกหรือไม่ (all elephant):  □ ไม่ใช่ (no)  □ ใช่ (yes)  ชนิดของยา (please specify deworming drug)........................................................  8.4 ความถี่การอาบน้ำช้างต่อวัน (frequency of showing per day)  จำนวนครั้ง (times)………………………(times)  ช่วงเวลา (when)…………………………...  ช้างอาบเองหรือไม่ (shower by itself, or not?)  □ ไม่ใช่ (no)  □ ใช่ (yes)  8.5 ปัญหาสุขภาพที่พบ (health problems)  □ไม่มี (no)  □ มี (yes)โปรดระบุ (please specify)………………………………………….  8.6 ลักษณะพฤติกรรมที่เปลี่ยนไป (abnormal behavior)  ………………………………………………………………………………………………………………………………………………………………………… |  |
| 9. โปรแกรมผสมพันธุ์ (breeding management) | 9.1 มีช้างพ่อแม่พันธุ์หรือไม่ (breeder):  □ ไม่มี (no)  □ มี (yes)  จำนวน (number): □ พ่อพันธุ์ (bull).....................เชือก (elephants)  □ แม่พันธุ์ (dam).....................เชือก (elephants)  9.2 ช่วงนี้มีโปรแกรมการผสมพันธุ์หรือไม่ อย่างไร  (In this period, Do they have breeding program? How?)  …………………………………………………………………………………………………………………………………………………………………………  9.3 มีการนำช้างพ่อแม่พันธุ์จากที่อื่นมาผสมในปางหรือไม่  (breeders from other camps come to breed at the camp)  □ ไม่มี (no)  □ มี (yes)  9.4  มีการนำช้างพ่อแม่พันธุ์ในปางไปผสมที่ปางอื่นหรือไม่  (breeders of the camp go to breed at other camps)  □ ไม่มี (no)  □ มี (yes)  9.5 โอกาสในการผสม (mating possibility) (น้อย/ปานกลาง/มาก)  ……………………………………………………………………………………..  9.6 โอกาสที่จะได้อยู่รวมฝูง (พฤติกรรมทางสังคม) (socialization in herd)  …………………………………………………………………………………….. | 9.1 มีช้างพ่อแม่พันธุ์หรือไม่ (breeder):  □ ไม่มี (no)  □ มี (yes)  จำนวน (number): □ พ่อพันธุ์ (bull).....................เชือก (elephants)  □ แม่พันธุ์ (dam).....................เชือก (elephants)  9.2 ช่วงนี้มีโปรแกรมการผสมพันธุ์หรือไม่ อย่างไร  (In this period, Do they have breeding program? How?)  …………………………………………………………………………………………………………………………………………………………………………  9.3 มีการนำช้างพ่อแม่พันธุ์จากที่อื่นมาผสมในปางหรือไม่  (breeders from other camps come to breed at the camp)  □ ไม่มี (no)  □ มี (yes)  9.4  มีการนำช้างพ่อแม่พันธุ์ในปางไปผสมที่ปางอื่นหรือไม่  (breeders of the camp go to breed at other camps)  □ ไม่มี (no)  □ มี (yes)  9.5 โอกาสในการผสม (mating possibility) (น้อย/ปานกลาง/มาก)  ……………………………………………………………………………………..  9.6 โอกาสที่จะได้อยู่รวมฝูง (พฤติกรรมทางสังคม) (socialization in herd)  …………………………………………………………………………………….. |  |
| 10. การจัดการช้างตกมัน  (musth management) | 10.1 ช้างในปางมีการตกมันหรือไม่ กี่ตัว  (Is there musth elephant in the camp? How many? )  □ ไม่มี (no)  □ มี (yes)…………………………….ตัว  10.2 มีการปรับโภชนาการให้ช้างตกมันหรือไม่   (Do you change the nutrition for musth elephant?)  □ ไม่มี (no)  □ มี (yes) โปรดระบุ (please specify)...................................................................  10.3 แหล่งน้ำ (water source)  …………………………………………………………………………………….  10. 4 วิธีการให้น้ำ (type of water drinking)  □ มีแหล่งน้ำให้กินได้ไม่จำกัด (ad libitum)  □ ควาญเอาให้กิน (by mahout)  10.5 การควบคุมช้างตกมัน (musth control)  10.5.1 พื้นที่แยกเฉพาะ (private zone)  □ ไม่มี (no)  □ มี (yes) โปรดระบุ (please specify).....................................................................  10.5.2 จำนวนตัวช้างต่อพื้นที่ (number of elephant each private zone)  …………………………………………..ตัวต่อพื้นที่ (elephant per zone)  10.5.3 มีการผูกโซ่หรือไม่ (chain)  □ ไม่มี (no)  □ มี (yes): ความยาวโซ่ (length of chain)..............................เมตร (meters)  10.5.4 อุปกรณ์ที่ใช้ประกอบ (other equipment)  …………………………………………………………………………………. | 10.1 ช้างในปางมีการตกมันหรือไม่ กี่ตัว  (Is there musth elephant in the camp? How many? )  □ ไม่มี (no)  □ มี (yes)…………………………….ตัว  10.2 มีการปรับโภชนาการให้ช้างตกมันหรือไม่   (Do you change the nutrition for musth elephant?)  □ ไม่มี (no)  □ มี (yes) โปรดระบุ (please specify).....................................................................  10.3 แหล่งน้ำ (water source)  …………………………………………………………………………………….  10. 4 วิธีการให้น้ำ (type of water drinking)  □ มีแหล่งน้ำให้กินได้ไม่จำกัด (ad libitum)  □ ควาญเอาให้กิน (by mahout)  10.5 การควบคุมช้างตกมัน (musth control)  10.5.1 พื้นที่แยกเฉพาะ (private zone)  □ ไม่มี (no)  □ มี (yes) โปรดระบุ (please specify)......................................................................  10.5.2 จำนวนตัวช้างต่อพื้นที่ (number of elephant each private zone)  …………………………………………..ตัวต่อพื้นที่ (elephant per zone)  10.5.3 มีการผูกโซ่หรือไม่ (chain)  □ ไม่มี (no)  □ มี (yes): ความยาวโซ่ (length of chain)..............................เมตร (meters)  10.5.4 อุปกรณ์ที่ใช้ประกอบ (other equipment)  …………………………………………………………………………………. |  |
| 11. โปรแกรมทำความสะอาด  (cleaning program) | 11.1 ความถี่เก็บอึ (frequency of defaces)  …………………………………………ครั้งต่อวัน (time per day)  มีการจัดการมูลช้างอย่างไร (elephant feces)  □ ทิ้ง (discard) โปรดระบุ (please specify)........................................................  □ ทำปุ๋ย (fertilizer)     □ มีผู้มารับซื้อ (sell)   □ เผา (burn)  11.2 ความถี่เก็บอาหารที่เหลือ (frequency of discarding food )  …………………………………………ครั้งต่อวัน (time per day)  มีการจัดการของเสียที่เหลือจากการให้อาหารช้างอย่างไร (waste from food)  □ ทิ้ง (discard) โปรดระบุ (please specify)...........................................................  □ ทำปุ๋ย (fertilizer)     □ มีผู้มารับซื้อ (sell)   □ เผา (burn)  11.3 ความถี่ทำความสะอาดพื้นคอก (frequency of cleaning the floor)  …………………………………………ครั้งต่อวัน (time per day) | 11.1 ความถี่เก็บอึ (frequency of defaces)  …………………………………………ครั้งต่อวัน (time per day)  มีการจัดการมูลช้างอย่างไร (elephant feces)  □ ทิ้ง (discard) โปรดระบุ (please specify)............................................  □ ทำปุ๋ย (fertilizer)     □ มีผู้มารับซื้อ (sell)   □ เผา (burn)  11.2 ความถี่เก็บอาหารที่เหลือ (frequency of discarding food )  …………………………………………ครั้งต่อวัน (time per day)  มีการจัดการของเสียที่เหลือจากการให้อาหารช้างอย่างไร (waste from food)  □ ทิ้ง (discard) โปรดระบุ (please specify)........................................................  □ ทำปุ๋ย (fertilizer)     □ มีผู้มารับซื้อ (sell)   □ เผา (burn)  11.3 ความถี่ทำความสะอาดพื้นคอก (frequency of cleaning the floor)  …………………………………………ครั้งต่อวัน (time per day) |  |
| 12. การจัดการควาญ (mahout management) | 12.1 จำนวนควาญช้างทั้งหมดในปาง  (number of mahout in the camp)..............................................คน (persons)  □ ควาญจริง (real mahout)........................................คน (persons)  □ ควาญสำรอง (alternate mahout)........................................คน (persons)  12.2 สัญชาติ (nationality)  ……………………………………………………………………………………..  12.3 อัตราส่วนควาญต่อช้าง (mahout: elephant ratio)  ……………………………………………………………………………………..  12.5 วันหยุด/วันลา (vacation)  ……………………………………………………………………………………. | 12.1 จำนวนควาญช้างทั้งหมดในปาง  (number of mahout in the camp)..............................................คน (persons)  □ ควาญจริง (real mahout)........................................คน (persons)  □ ควาญสำรอง (alternate mahout)........................................คน (persons)  12.1.1 สาเหตุจำนวนควาญลดลง (cause of decreasing mahout)  □ พักงาน(suspended temporary job)  □ ทำงานอื่นร่วมด้วย (do other jobs)  □ ลาออก (quit)  □ อื่นๆ (others)  12.2 การจัดการเมื่อควาญลดลง (How does camp manage when mahout was decreased?)  ……………………………………………………………………………………..  12.3 สัญชาติ (nationality)  ……………………………………………………………………………………..  12.4 อัตราส่วนควาญต่อช้าง (mahout: elephant ratio)  ……………………………………………………………………………………..  12.5 วันหยุด/วันลา (vacation)  ……………………………………………………………………………………. |  |
| 12. ความช่วยเหลือที่ได้รับ (helping) | - 1. ปางมีการได้รับความช่วยเหลือหรือไม่ (Does camp get helping, or not?)   □ ไม่มี (no)  □ มี (yes)   \| แหล่งที่มา (source of helping) \| สิ่งที่ได้รับ (What did you get help?) \| \| --- \| --- \| \|  \|  \|  - 1. ช่วงเวลาที่ได้รับ (when?)   ................................................................................................................................ | 12.1 ปางมีการได้รับความช่วยเหลือหรือไม่ (Does camp get helping, or not?)  □ ไม่มี (no)  □ มี (yes) □ มี (yes)   \| แหล่งที่มา (source of helping) \| สิ่งที่ได้รับ (What did you get help?) \| \| --- \| --- \| \|  \|  \|  - 1. ช่วงเวลาที่ได้รับ (when?)   ................................................................................................................................ |  |
| 13. ทัศนคติ (attitude) | 13.1 ก่อนหน้านี้ปางประสบปัญหาอะไรมาก่อนหน้านี้ไหม (in previous period, do you have any problem?)  …………………………………………………………………………………….  …………………………………………………………………………………….  …………………………………………………………………………………….  …………………………………………………………………………………….  …………………………………………………………………………………….  13.2 จุดประสงค์ของการเปิดปาง/แรงจูงใจในการทำงาน (motivation of work)  …………………………………………………………………………………….  …………………………………………………………………………………….  …………………………………………………………………………………….  13.3 แผนรับมือกับภาวะวิกฤตอื่นๆ หรือสถานการณ์ฉุกเฉินอย่างไร (Do you have any plan to cope with other crisis? )  …………………………………………………………………………………….  …………………………………………………………………………………….  …………………………………………………………………………………….  …………………………………………………………………………………….  …………………………………………………………………………………….  …………………………………………………………………………………….  …………………………………………………………………………………….  …………………………………………………………………………………….  …………………………………………………………………………………….  …………………………………………………………………………………….  …………………………………………………………………………………… | 13.1 แรงจูงใจในการทำงาน (motivation of work)  …………………………………………………………………………………….  …………………………………………………………………………………….  …………………………………………………………………………………….  13.2 ปางมีความคิดเห็นต่อโควิด-19 อย่างไรบ้าง และให้คะแนนความรุนแรง (what do you think about the COVID-19 crisis? Can you give the affected score?)  …………………………………………………………………………………….  …………………………………………………………………………………….  …………………………………………………………………………………….  …………………………………………………………………………………….  …………………………………………………………………………………….  13.3 ปางมีรูปแบบการรับมือต่อโควิดอย่างไร (How to cope with the COVID-19?)  …………………………………………………………………………………….  …………………………………………………………………………………….  …………………………………………………………………………………….  …………………………………………………………………………………….  …………………………………………………………………………………….  …………………………………………………………………………………….  …………………………………………………………………………………….  …………………………………………………………………………………….  13.4 หลังสถานการณ์การระบาดโควิด-19 ทางปางจะมีการจัดกิจกรรมเหมือนเดิมหรือแตกต่างจากเดิมไปอย่างไร (What activities will happen after the COVI)-19)  ……………………………………………………………………………………  ……………………………………………………………………………………  ……………………………………………………………………………………  ……………………………………………………………………………………  …………………………………………………………………………………… |  |
